# Synergistic effects of chemical and biochemical fertilization on yield enhancement and oil quality optimization in ‘Zard’ olive cultivars

**DOI:** 10.3389/fpls.2025.1455921

**Published:** 2025-01-23

**Authors:** Mohammad Saeed Tadayon, Ahmad Asgharzadeh, Seyed Majid Mousavi, Kobra Saghafi

**Affiliations:** ^1^ Soil and Water Research Department, Agricultural Research, Education and Extension Organization (AREEO), Shiraz, Iran; ^2^ Biology and Biotechnology Department, Soil and Water Research Institute, Agricultural Research, Education and Extension Organization (AREEO), Karaj, Iran; ^3^ Soil and Water Research Institute, Agricultural Research, Education and Extension Organization (AREEO), Karaj, Iran; ^4^ Biology and Biotechnology Department, Soil and Water Research Institue, Agricultural Research, Education and Extension Organization (AREEO), Karaj, Iran

**Keywords:** biological fertilizer, chemical fertilizer, oil quality, olive, plant nutrition

## Abstract

**Purpose:**

This research evaluates the combined impact of chemical and biological fertilizers on ‘Zard’ olive trees, aiming to reduce chemical dependency, enhance fertilizer efficiency, and improve nutritional value, yield, and oil quality from 2020 to 2023.

**Method:**

A factorial design within a randomized complete block was used, focusing on the first factor, soil chemical fertilizer application (CF) at three levels, 100% (CF100), 75% (CF75), and 50% (CF50) of the fertilizer requirement as determined by soil testing. This was coupled with foliar applications of 20-20-20 NPK fertilizer with micronutrients. The second factor, biological fertilizer application (BF), also comprised three levels: BF0 (control), soil-applied organic fertilizer without biological agents; BF1, which included a soil application of an organic fertilizer mix, mycorrhizal fungi, and the beneficial bacteria *Bacillus subtilis* and *Pseudomonas fluorescens*, supplemented with fulvic acid and amino acids; and BF1+BFF, where trees were treated with both soil and foliar applications of the aforementioned bacterial species, fulvic acid, and amino acids.

**Results:**

The CF100+BF1+BFF treatment significantly increased fruit length (31.14%), diameter (41.61%), flesh thickness (30.48%), fresh weight (38.76%), dry weight (55.68%), and yield per tree (27.00%) compared to the control (CF100+BF0). Principal Component Analysis (PCA) identified CF100+BF1+BFF, CF75+BF1+BFF, and CF50+BF1+BFF as superior treatments for fruit characteristics, while CF50+BF1+BFF excelled in oil quality indicators.

**Conclusion:**

The study recommends the CF75+BF1+BFF and CF50+BF1+BFF treatments for concurrent improvements in fruit and oil quality. The combined use of biological fertilizers with reduced chemical fertilizers is considered the superior and optimal approach for fertilizing ‘Zard’ cultivar olive orchards.

## Introduction

Olive trees (*Olea europaea* L.) are recognized for their potential to counteract the adverse effects of climate change, offering a sustainable alternative to crops that are more vulnerable to drought in arid and semi-arid regions (Nteve et al., 2024). In Iran, the expansion of olive orchards has been noteworthy over recent decades. Currently, olive cultivation occupies an area of approximately 100,000 hectares, with an annual yield of 70 to 80 thousand tons of olives, from which 5,000 to 5,500 tons of oil are extracted (FAO, 2023). The formulation of effective horticultural strategies is imperative to boost productivity and ensure the integrity of both the fruit and the oil (Brito et al., 2019). Nutritional management is critical in enhancing the overall performance of olive trees (Jiménez-Moreno and Fernández-Escobar, 2017; Tadayon and Hosseini, 2023). Improving nutrient bioavailability in proximity to the olive roots is vital for augmenting plant metabolism and consequently, the quality of the fruit (Zipori et al., 2020). The interplay among soil, roots, and microorganisms constitutes a dynamic and integral network. This network, in synergy with chemical fertilizers, is instrumental in maintaining soil fertility. Nutrient deficiencies can impede yield, yet the overuse of chemical fertilizers may lead to a decline in soil microbial diversity, reduced soil fertility, and topsoil degradation, potentially causing soil compaction, as reported by Herrmann and Lesueur (2013). In contrast, incorporating biofertilizers can lessen reliance on chemical fertilizers and mitigate environmental risks. The strategic use of biofertilizers supports sustainable agriculture and contributes to the yield of high-quality olive crops, as evidenced by Ben Salah et al. (2018).

Recent research indicates that biological fertilizers, along with beneficial microorganisms, collectively referred to as microbial flora, substantially enhance the efficacy of both chemical and organic fertilizers. This enhancement is most pronounced when biological fertilizers complement chemical fertilizers and, on rare occasions, can even replace them (Zipori et al., 2020). Biological fertilizers are consistently shown to increase soil microbial diversity, alleviate soil-related stressors, contribute to overall biodiversity, and augment the bioavailability of nutrients within the soil matrix (Bhattacharyya et al., 2020; Melloni and Cardoso, 2023).

Plant growth-promoting bacteria (PGPB) are crucial biotic constituents of soil ecosystems, playing an indispensable role in the sustainable cultivation of agricultural and non-agricultural plant species. The absence of PGPB from the soil ecosystem would markedly hinder plant growth and development (Ben Salah et al., 2018; Jacob and Paranthaman, 2023). Additionally, PGPB application provides a defensive mechanism against phytopathogens and other deleterious organisms, thereby facilitating bioremediation processes and protecting various soil horizons (Ammar, 2024).

Arbuscular mycorrhizal fungi (AMF) have been observed to enhance critical physiological parameters, including the photosynthetic rate and mineral nutrient profile of olive trees (Tekaya et al., 2017; Boutaj et al., 2020). Moreover, the supplemental introduction of AMF has been linked to improved survival rates and ecophysiological performance of olive trees, particularly in arid environments (Ennajeh and Ouledali, 2024).

Nutrient deficiencies, particularly under drought conditions, can severely impact plant growth due to hindered nutrient absorption, translocation, and redistribution within the plant, especially in soils with low organic matter and moisture content (Marschner, 2011; Zipori et al., 2020). The application of organic matter, in conjunction with biofertilizers, by enhancing soil moisture retention, can optimize plant metabolic processes and increase nutrient use efficiency (Herrmann and Lesueur, 2013; García-Fraile et al., 2015; Raimi et al., 2017). Biofertilizers provide a wide spectrum of absorbable nutrients and growth promoters for plants, thus establishing a foundation for increased productivity per unit area (Bhattacharyya et al., 2020; Bizos et al., 2020; Jacob and Paranthaman, 2023; Melloni and Cardoso, 2023). Alowaiesh et al. (2023) evaluated the effectiveness of organic and bio-fertilizers on olive trees cultivated in low-fertility sandy soils. The study revealed that the application of goat manure, combined with nitrogen-fixing bacteria, significantly enhanced vegetative growth, yield, and fruit quality.

The foliar application of biological fertilizers, even in minimal quantities, effectively augmented the levels of antioxidants in the leaves and fruits of various olive cultivars grown in calcareous soils (Maksoud et al., 2009). This elevation in antioxidant content within olive fruits is vital for human health. Dabbaghi et al. (2018) showed that foliar application of biofertilizers on olive trees enhances both the quantitative and qualitative characteristics of the fruit. Furthermore, Sotiropoulos et al. (2024) conducted research on mature olive trees, revealing that foliar application of a liquid organic product containing fulvic and humic acids significantly impacted fruit yield and oil production, particularly in alkaline soils.

The foliar application of amino acids, in conjunction with fulvic acids, leverages the high absorption and remobilization of nutrient elements through the plant’s aerial system, potentially leading to maximal plant growth (Han et al., 2024). Specific amino acids, such as cysteine and phenylalanine, increase the antioxidant metabolism and the activity of resistance enzymes when applied as a foliar treatment (Trovato et al., 2021). The foliar application of amino acids with biofertilizers, such as *Bacillus amyloliquefaciens*, has been shown to improve crop yield. The addition of these beneficial microbes may also play a role in altering the composition of the leaf microbiota, thereby enhancing plant health (Wang et al., 2019). It appears that the combination of amino acids and plant growth-promoting bacteria can effectively increase root volume and crop production (Freitas and Silva, 2022).

Considering these factors, the present study aims to explore the impact of integrated nutrition, utilizing both biological and chemical fertilizers, on diminishing the reliance on chemical fertilizers. The experiment also seeks to assess the enhancement of plant nutrient status and the subsequent effects on the yield and oil production quality and quantity in olive trees (*Olea europaea* L. cv ‘Zard’).

## Materials and methods

### Site description

The study was conducted in a commercial orchard of 19-year-old, own-rooted olive trees (*Olea europaea* L. cv. ‘Zard’), planted at a density of 238 trees per hectare. The planting arrangement consisted of 6 m between trees within rows and 7 m between rows, with a modified central leader training system. The orchard was equipped with a drip irrigation system, and irrigation was scheduled based on 70% of Class A pan evaporation, applied at 4-day intervals throughout the experiment. The orchard is situated at coordinates 29°36’40.2192”N, 52°47’39.988”E, at an altitude of 1624 meters, to the east of Shiraz in Fars Province, Iran. This area is known for its semi-arid climate, characterized by hot summers, relatively cold winters, and an average annual temperature fluctuating between 17 to 19°C. The research spanned four consecutive years, from 2020 to 2023. In February 2019, before the implementation of the experimental treatments, sample of irrigation water and composite soil were collected from two depths: 0-30 cm and 30-60 cm beneath the canopy’s edge. The water and soil sample’s characteristics were analyzed using the methods outlined by Estefan (2013), at the analytical laboratory of the Soil and Water Research Department, Fars Agricultural and Natural Resources Research and Education Center, Zarghan, Iran.

The chemical characteristics of the irrigation water sample in the experimental olive (*Olea europaea* L. cv. ‘Zard’) orchard are provided in **Table 1**. The irrigation water quality is generally within acceptable limits for olive cultivation. However, the slightly alkaline pH and the high sodium adsorption ratio (SAR) value suggest that there may be a long-term risk of soil alkalinity and sodium accumulation. The soil at the experimental site is classified as calcareous with a silt loam texture. The moderate salinity and alkaline pH are within the tolerance range of olive trees. It contains a low level of macro- and micronutrients and organic carbon (**Table 2**).

**Table 1:** The chemical characteristics of the irrigation water sample in the experimental olive (*Olea europaea* L. cv. ‘Zard’) orchard.

Electrical Conductivity (EC) (dS/m)	pH	Carbonate (meq/L)	Bicarbonate (meq/L)	Chloride (meq/L)	Sulfate (meq/L)	Total Magnesium and Calcium (meq/L)	Boron (mg/L)	Sodium (meq/L)	Total Cations (meq/L)	Sodium Adsorption Ratio (SAR)
1.85	8.2	0.69	2.57	1.26	0.43	0.53	0.75	4.43	4.96	8.69

**Table 2:** Soil physico-chemical characteristics in the experimental olive (*Olea europaea* L. cv. ‘Zard’) orchard.

Soil depth (cm)	Bulk density (g/cm^3^)	Electrical Conductivity (dS/m)	pH	Total Nutralizing Value	Organic Carbon	P	K	Mg	Ca	Fe	Zn	Mn	Cu	B	Soil Texture
				%				(mg/kg)							
0-30	1.25	2.48	8.8	45.2	0.78	5.5	125.5	211.2	522.3	3.2	0.59	4. 4	0.33	1.1	Silt Loam
30-60	1.28	2.36	8.9	47.7	0.62	2.9	124.4	213.6	537.1	3.3	0.54	4.3	**0.32**	**1.2**	**Silt Loam**

### Experimental factors

In February 2019, 120 olive trees (*Olea europaea* L. cv. ‘Zard’), exhibiting similar canopy sizes, were systematically selected and labeled in accordance with the experimental design. All cultural and management practices were uniformly applied across the trees, with the exception of the specified treatment variables. The experimental design was factorial, implemented in a randomized complete block design with two factors. The first factor, Soil Chemical Fertilizer Application (CF), was tested at three levels, 100% (CF100), 75% (CF75), and 50% (CF50) of the fertilizer requirement as determined by soil testing. This was coupled with foliar applications of a balanced 20-20-20 NPK fertilizer enriched with micronutrients. The second factor, Biological Fertilizer Application (BF), also comprised three levels: BF0 (control), where trees received only soil-applied organic fertilizer without biological agents; BF1, which included a soil application of an organic fertilizer mix, mycorrhizal fungi, and the beneficial bacteria *Bacillus subtilis* and *Pseudomonas fluorescens*, supplemented with fulvic acid and amino acids; and BF1+BFF, where trees were treated with both soil and foliar applications of the aforementioned bacterial species, fulvic acid, and amino acids. The experimental design was structured with four replications per treatment, each comprising five trees. The foliar application of the bacterial consortium (*Bacillus subtilis* and *Pseudomonas fluorescens*), fulvic acid, and amino acids, in conjunction with the 20-20-20 chemical fertilizer containing micronutrients at a 0.3% concentration, was performed twice: once during the flower initiation phase, at the 51-55 stage on the BBCH scale, as described by Sanz-Cortés et al. (2002), which precedes full bloom. The second application was timed to align with the beginning of flower induction, at the 71-75 stage on the BBCH scale, coinciding with the commencement of fruit stone hardening. The complete 20-20-20 NPK fertilizer (Woprofert Netherlands) contains ammonium nitrogen (NH_4_^+^) at 3.9%, nitrate nitrogen (NO_3_^-^) at 5.9%, and urea at 10.2%. It provides water-soluble phosphorus (P_2_O_5_) at 20%, water-soluble potassium (K_2_O) at 20%, and essential micronutrients such as iron (Fe-EDTA) at 260 ppm, copper (Cu-EDTA) at 75 ppm, manganese (Mn-EDTA) at 320 ppm, zinc (Zn-EDTA) at 230 ppm, and boron (as boric acid, H_3_BO_3_) at 100 ppm.

Based on soil test results, a 100% chemical fertilization regimen was established, comprising the application of Urea (46-0-0, 750 g per tree), Potassium sulfate (0-0-53 + 17S, 900 g per tree), and Diammonium phosphate (DAP) (18-46-0, 750 g per tree), along with Iron chelate (300 g per tree of Sequestrene 138-Fe EDDHA 6%). These fertilizers were applied at three pivotal growth stages: the onset of flush growth, two-thirds into the flush growth period, and during early fruit development in June. Additionally, Zinc sulfate (33% Zn, 15% S, 300 g per tree), manganese sulfate (31% Mn, 18% S, 300 g per tree), and copper sulfate (25.5% Cu, 12.5% S, 150 g per tree) were administered in early spring to all olive trees. The application of chemical fertilizers to the soil was performed via fertigation, utilizing a fertilizer tank.

In the preparation of the biological fertilizer, a bacterial inoculant in liquid form was utilized. The characteristics of the bacterial population present in the commercial inoculum, which was applied in the biological fertilizer treatment, are detailed in **Table 3**. Additionally, mycorrhizal fungi were cultivated in solid form and subsequently combined with the bacterial inoculant, amino acids, and fulvic acid to create a comprehensive biological treatment.

**Table 3:** Population of counted bacteria in the commercial inoculum used in the biological fertilizer treatment.

Type of Bacteria	Population
*Pseudomonas fluorescens* (BioNik-M)	1.7 × 10^8^ Cells ml^-1^
*Bacillus subtilis* (BioNik-M)	2 × 10^8^ Cells ml^-1^
Population of mycorrhizal powder inoculum (Arbuscular mycorrhizal fungi AMF, *Rhizophagus irregularis*, BioNik-M)	10^5^ Cells gr^-1^ Propagule

The method of soil application for the organic and biological fertilizer treatment involved channel fertilization at a depth of 50 cm beneath the canopy edge (in late winter), supplemented by 45 kg of decomposed cow manure. The quantity of combined biological fertilizer used for each tree included 500 g of mycorrhizal fungi and 250 mL of each bacterial inoculant (totaling 500 mL for two bacterial species), along with 100 mL of amino acids and fulvic acid for soil application. Additionally, foliar spraying of the biological fertilizer, excluding mycorrhizal fungi but incorporating a combination of two bacterial species at 250 mL each, plus 100 mL of amino acids and fulvic acid, was conducted with sufficient water to ensure complete wetting of each tree.

### Leaf tissue analysis

Fully developed leaves for each season were collected around late August, which is approximately three weeks following the application of experimental treatments. These leaves were taken from the middle section of non-fruit-bearing branches. The collected samples were then washed with distilled water, oven-dried at 70˚C and analyzed in accordance with the methodologies outlined by Estefan (2013). This analysis was conducted at the analytical laboratory of the Soil and Water Research Department, located at the Fars Agricultural and Natural Resources Research and Education Center in Zarghan, Iran. Briefly, nutrient elements such as total nitrogen were measured using the micro-Kjeldahl method with a Kjeltec auto analyzer (Tecator, 1030). Phosphorus was quantified by colorimetry using a Pharmacia spectrophotometer (Pharmacia LKB, Novaspec II), potassium by a Jenway flame photometer (Jenway, PFP7), and the concentrations of calcium and magnesium were determined by complexometric titration. Trace elements, including zinc, manganese, iron, copper, were quantified by atomic absorption spectroscopy using a PerkinElmer instrument (Perkin Elmer 1100 B), following sample ashing in a furnace and digestion with 0.1 N hydrochloric acid. Boron was determined by the azomethine-H method.

### Quantitative and qualitative characteristics of olive fruit and oil

Olive fruits were harvested when 50% of the olive fruit displayed partial or total purple color; this was equivalent to a maturity index of 7 for at least 50% of the fruit harvested. Harvesting was performed using electric combs (Olivium, Pellenc, France), which facilitated the detachment of the fruit onto plastic nets during the commercial harvest period in October. The yield was then gathered, weighed, and the total fruit yield per tree was documented using a scale. Subsequently, a representative 3 kg sample was randomly selected for comprehensive analysis of the fruit’s oil yield and quality.

The fresh weight of one hundred randomly selected fruit samples per replicate was accurately measured using a digital balance with a sensitivity of 0.001 g. The dry weight was determined post-dehydration of the samples for 48 hours at 80°C, and the average individual fruit fresh and dry weight was subsequently calculated. The dimensions of the fruit, specifically length and width, were measured for one hundred random fruit samples per replicate using digital calipers.

Oil extraction was conducted using a laboratory-scale olive mill according to Juliano et al. (2023) method, where the olive fruits were crushed at a consistent speed of 3000 rpm using a hammer mill (FP HP 40 model, Gruppo Pieralisi). Approximately 700 g of the resulting paste from the hammer mill was subjected to malaxation at 50 rpm for 30 minutes at a controlled temperature of 28°C. Subsequently, the paste was centrifuged at 1400× g for 2 minutes, followed by a secondary centrifugation of the liquid phase at 3500 rpm for 10 minutes using a Fisher Scientific Accuspin 3R Centrifuge. The extracted oil was meticulously separated and preserved in 100 mL dark glass bottles at -20°C until further analysis.

To ascertain the fruit water content, approximately 30 g aliquot of the resulting paste was transferred to a pre-weighed Petri dish, dried in a fan-forced oven at 80°C for 24 hours until a constant weight was reached, and subsequently allowed to cool to room temperature in a desiccator. The dry weight of the sample was recorded, and the water content was calculated as a percentage of the total fruit weight. The oil content of the fruit was determined per gram of dry weight using the Soxhlet extraction method, with hexane as the extraction solvent (Trolles-Cavalcante et al., 2021). Assessments of fatty acids (FFA), peroxide index and specific extinction coefficients K_232_, K_270_, K_262_, K_268_, K_274_ and DeltaK (ΔK) in olive oil were carried out according to the International Olive Council methods of analysis (IOC, 2019).

Free Fatty Acids (FFA) in olive oil are analyzed by gas chromatography (GC) method. Olive oil samples were stored at -20°C prior to analysis. Upon thawing at room temperature, 0.05 g of each sample was transferred into a screw-capped test tube with Teflon seals. Analyses were performed in duplicate, with the addition of either 0.5 ml of C17:0 (a saturated fatty acid with a 17-carbon chain) in toluene solution (0.75 mg/ml) or 0.5 ml of C21:0 in toluene solution (1.25 mg/ml) as internal standards. To each tube, 0.5 ml of toluene and 1.7 ml of methanol: toluene (4:1 v/v) were added—these solvents were utilized for lipid extraction prior to GC analysis. The tubes were then vortexed for homogenization. The methylation of the extracted lipids was facilitated by adding 0.25 ml of acetyl chloride, followed by incubation at 100°C for 1 hour. Post-incubation, the samples were allowed to cool to room temperature. Subsequently, 5 ml of 5% potassium chloride was added, and the samples were centrifuged at 1000 rpm for 6 minutes using a Fisher Scientific accuspin 3R centrifuge (Saint-Laurent, Canada). The supernatant was carefully decanted for the analysis of fatty acid methyl esters (FAMEs) by gas chromatography (GC). FAMEs were analyzed using an Agilent GC System (model 5975). Purified helium served as the carrier gas, maintained at a head pressure of 109.9 kPa and a column flow rate of 0.43 mL/min. The split ratio was set at 89.8, with an injector temperature of 250°C. Detection and quantification of FAME peaks were conducted using a flame ionization detector (FID) at 250°C. For the GC injection, 1 μL of the sample was introduced at an initial oven temperature of 50°C, which was maintained for 1 minute. The temperature was then increased at a rate of 2°C/min to 188°C, held for 10 minutes, followed by a further increase at 2°C/min to the final temperature of 240°C, which was sustained for 44 minutes. The total run time amounted to 150 minutes. Peak identification was achieved using a 37-component FAME standard (Supelco, FAME mix C4-C24, 100 mg). The area of each individual peak was expressed as a percentage of the total peak area.

Peroxide value (PV) is a common method for evaluating oxidation in fats and oils which affects their quality, particularly in relation to the off-flavour and rancidity development (Gilbraith et al., 2021). High temperature, exposure to visible or diffused light and oxygen, contact with metal surfaces (e.g., copper), physical, disease and pest damage to fruit and delays between harvest and processing time can increase the risk of oxidation of olive oils (Kiritsakis et al., 2020).

Specific extinction coefficients (values) K_232_, K_270_, K_262_, K_268_, K_274_ and DeltaK (ΔK) in olive oil are determined by a spectrophotometric (Pharmacia LKB, Novaspec II) method. A sample of olive oil is dissolved in a specified solvent (iso-octane or cyclohexane) to create a solution with a concentration of 1% (m/V). The absorbance of the solution at the specified wavelengths (232 nm and either 268 nm in iso-octane or 270 nm in cyclohexane) with reference to pure solvent. The specific extinctions at 232 nm and 268/270 nm are calculated for the 1% concentration in a 10 mm cell. Specific extinction coefficients at those wavelengths are pivotal in determining the oxidation status of olive oil. During the oxidation process, conjugated peroxides, primarily hydroperoxides, emerge as initial oxidation products, leading to characteristic absorption peaks at 232 nm within the ultraviolet (UV) spectrum. These hydroperoxides subsequently decompose into secondary oxidation products, such as aldehydes and ketones, which exhibit absorption around 270 nm. The absorbance readings at 262 nm, 268 nm, and 274 nm facilitate the calculation of ΔK (ΔK = K_268_ − ((K_262_ + K_274_)/2)), which is instrumental in categorizing the oxidation levels of olive oil (IOC, 2019; Frangipane et al., 2023).

Extra virgin olive oil is classified with a maximum free acidity, expressed as free oleic acid, of 0.8 g per 100 g, a peroxide value lower than 20, a K_232_ lower than 2.50, a K_268_ or K_270_ of lower than 0.22, and a ΔK lower than 0.01 (IOC, 2019).

### Statistical analysis

The percentage data, prior to statistical analysis, were normalized using the arcsine square root transformation. The combined analysis of variance (ANOVA) of the data from the four-year experiment was conducted using SAS software (Alonso et al., 2016), and the comparison of treatment means was performed with the Least Significant Difference (LSD) test. The correlations were calculated using Microsoft Excel 2016 (Jackson, 2016). The relationship between various measured parameters was analyzed by principal component analysis (PCA) using Minitab 14 software (Wild, 2005).

## Results

### Leaf nutrient content

In this study, we conducted a combined analysis of variance (ANOVA) on the dataset collected over a four-year period. This analysis allowed us to isolate the effect of time (year) and the interaction between time and treatment variables. Following this, we assessed both the simple and interaction effects of experimental factors on the measured traits. These effects were rigorously compared and scrutinized to understand their impact on the experimental outcomes.

**Tables 4** and **5** illustrate that varying levels of chemical fertilizers contained soil-applied chemical fertilizers, in conjunction with foliar sprays, significantly affected the nutrient element concentrations in the leaves of olive trees. In the Zard olive cultivar, the highest concentrations of macro and micronutrients were observed at the CF100 (control) and CF75 treatment levels, respectively. These levels, with the exception of the leaf iron (Fe) concentration, were categorized within the same statistical group. This indicates a potential for reducing chemical fertilizer inputs. However, reducing the chemical fertilizer to 50% (CF50) significantly decreased nutrient concentrations, dropping below the sufficiency levels for nitrogen (N), phosphorus (P), potassium (K), iron (Fe), manganese (Mn), zinc (Zn), and copper (Cu), which could lead to deficiencies. Notably, the leaf boron (B) concentration across the three distinct soil application levels, coupled with the foliar spray of chemical fertilizers, did not differ statistically, as evidenced in **Table 5**.

**Table 4:** The simple and interactive effects of chemical and biological fertilizers on the leaf macronutrient concentration in olive trees (*Olea europaea* L., cv. ‘Zard’).

Treatment	N	P	K	Ca	Mg	S
	(%)					
I- Chemical fertilizers						
CF100 (control)	2.38 ± 0.22a	0.16 ± 0.02a	1.54 ± 0.16a	1.85 ± 0.17a	0.45 ± 0.06a	0.20 ± 0.02a
CF75	2.11 ± 0.22a	0.14 ± 0.01a	1.46 ± 0.15a	1.64 ± 0.15ab	0.43 ± 0.03a	0.17 ± 0.02a
CF50	1.42 ± 0.15b	0.09 ± 0.01b	0.97 ± 0.11b	1.49 ± 0.16b	0.31 ± 0.04b	0.12 ± 0.01b
**F. test**	**	**	**	*	*	**
II- Biological fertilizers						
BF0 (control)	1.66 ± 0.13b	0.09 ± 0.01b	1.05 ± 0.10b	1.45 ± 0.13a	0.36 ± 0.03a	0.13 ± 0.01b
BF1	2.00 ± 0.23a	0.13 ± 0.01a	1.38 ± 0.12a	1.68 ± 0.14a	0.41 ± 0.04a	0.17 ± 0.02a
BF1+BFF	2.24 ± 0.24a	0.16 ± 0.02a	1.54 ± 0.13a	1.84 ± 0.15a	0.42 ± 0.04a	0.20 ± 0.02a
**F. test**	**	**	*	**ns**	**ns**	**
Interaction						
CF100+BF0 (Control)	2.04 ± 0.23bcd	0.12 ± 0.01c	1.26 ± 0.17bc	1.56 ± 0.13cd	0.41 ± 0.04abc	0.16 ± 0.02cd
CF100+BF1	2.41 ± 0.22ab	0.16 ± 0.02ab	1.61 ± 0.14a	1.89 ± 0.17ab	0.48 ± 0.05a	0.21 ± 0.02ab
CF100+ BF1+BFF	2.68 ± 0.25a	0.19 ± 0.02a	1.75 ± 0.13a	2.10 ± 0.22a	0.45 ± 0.05ab	0.23 ± 0.02a
CF75+BF0	1.83 ± 0.19c	0.09 ± 0.01d	1.15 ± 0.14cd	1.46 ± 0.13cd	0.43 ± 0.04ab	0.13 ± 0.01de
CF75+BF1	2.14 ± 0.24bc	0.15 ± 0.02abc	1.54 ± 0.12ab	1.67 ± 0.14bc	0.42 ± 0.05ab	0.18 ± 0.02bc
CF75+ BF1+BFF	2.37 ± 0.22ab	0.17 ± 0.02a	1.68 ± 0.15a	1.79 ± 0.15ab	0.45 ± 0.05ab	0.21 ± 0.02ab
CF50+BF0	1.12 ± 0.17f	0.06 ± 0.01e	0.75 ± 0.09e	1.34 ± 0.16d	0.23 ± 0.03d	0.09 ± 0.01e
CF50+BF1	1.45 ± 0.13e	0.08 ± 0.01de	0.98 ± 0.12d	1.49 ± 0.13cd	0.34 ± 0.02c	0.11 ± 0.01e
CF50+ BF1+BFF	1.68 ± 0.19de	0.13 ± 0.01bc	1.18 ± 0.10cd	1.63 ± 0.15bcd	0.37 ± 0.03bc	0.16 ± 0.02cd
**F. test**	**	**	**	**	*	**
**Sufficient (%)**	**1.50 – 2.00**	**0.10 – 0.30**	**> 0.80**	**> 1.0**	**> 0.10**	**0.1-0.25**

The data are presented as the main effect means with their corresponding Standard Error (SE). Within the same column, means annotated with identical letters do not exhibit statistically significant differences as determined by the Least Significant Difference (LSD) test. Significance levels are denoted by * (P ≤ 0.05) and ** (P ≤ 0.01) based on the F-test; ‘ns’ indicates a non-significant result. Chemical Fertilizer Application (CF), was tested at three levels, 100% (CF100), 75% (CF75), and 50% (CF50) of the fertilizer requirement as determined by soil testing. This was coupled with foliar applications of a balanced 20-20-20 NPK fertilizer enriched with micronutrients. Biological Fertilizer Application (BF), also comprised three levels: BF0 (control), where trees received only soil-applied organic fertilizer without biological agents; BF1, which included a soil application of an organic fertilizer mix, mycorrhizal fungi, and the beneficial bacteria Bacillus subtilis and Pseudomonas fluorescens, supplemented with fulvic acid and amino acids; and BF1+BFF, where trees were treated with both soil and foliar applications of the aforementioned bacterial species, fulvic acid, and amino acids.

Bold values: Olive leaf nutrients sufficient range according to (Kailis and Harris, 2007; Fernández-Escobar et al., 2009; Ali, 2023). Olive leaf tissue analysis from sub terminal leaflets of current year’s non-fruit bearing growth.

**Table 5:** The simple and interactive effects of chemical and biological fertilizers on the leaf micronutrient concentration in olive trees (*Olea europaea* L., cv. ‘Zard’).

Treatment	Fe	Mn	Zn	Cu	B
	(mg kg^-1^)				
I- Chemical fertilizers					
CF100 (control)	153.0 ± 16.1a	42.08 ± 4.71a	9.81 ± 0.83a	4.33 ± 0.52a	36.77 ± 3.95a
CF75	127.7 ± 13.9b	37.97 ± 3.60a	7.93 ± 0.74a	3.81 ± 0.41a	35.06 ± 4.11a
CF50	94.2 ± 9.7c	29.75 ± 2.58b	5.23 ± 0.62b	2.80 ± 0.30b	34.80 ± 3.12a
**F. test**	**	**	**	**	**ns**
II- Biological fertilizers					
BF0 (control)	98.3 ± 7.8c	30.14 ± 2.89b	5.45 ± 0.51c	3.30 ± 0.34b	33.33 ± 3.69ab
BF1	124.2 ± 13.1b	35.90 ± 3.24b	7.13 ± 0.67b	3.04 ± 0.25b	31.81 ± 3.54b
BF1+BFF	152.4 ± 14.5a	43.75 ± 4.25a	10.39 ± 1.18a	4.60 ± 0.39a	41.49 ± 4.81a
**F. test**	**	**	**	**	**ns**
Interaction					
CF100+BF0 (Control)	122.8 ± 11.5cd	35.61 ± 3.80cd	8.64 ± 0.82c	4.24 ± 0.45ab	34.32 ± 3.22bcd
CF100+BF1	142.3 ± 15.0bc	40.68 ± 4.16bc	9.32 ± 0.97bc	3.65 ± 0.41bc	32.49 ± 3.34d
CF100+ BF1+BFF	193.9 ± 18.2a	49.95 ± 5.05a	11.48 ± 0.95a	5.10 ± 0.48a	43.50 ± 4.16a
CF75+BF0	98.1 ± 10.4e	31.71 ± 3.12de	5.32 ± 0.61d	3.26 ± 0.31c	32.57 ± 3.26d
CF75+BF1	136.7 ± 12.9bcd	37.63 ± 3.19bcd	7.71 ± 0.81c	3.58 ± 0.38bc	31.25 ± 3.28d
CF75+ BF1+BFF	148.4 ± 13.5b	44.57 ± 4.11ab	10.76 ± 0.97ab	4.59 ± 0.43a	41.37 ± 4.02ab
CF50+BF0	74.0 ± 7.8f	23.11 ± 2.64f	2.40 ± 0.35e	2.40 ± 0.27d	33.10 ± 3.47cd
CF50+BF1	93.7 ± 9.5e	29.39 ± 2.23e	4.36 ± 0.48d	1.89 ± 0.21e	31.70 ± 3.63d
CF50+ BF1+BFF	114.9 ± 12.1de	36.74 ± 3.24bcd	8.94 ± 0.74c	4.12 ± 0.42b	39.61 ± 3.75abc
**F. test**	**	**	**	**	**
**Sufficient (mg kg^-1^)**	**> 90**	**> 20**	**> 10**	**4-9**	**19 – 150**

The data are presented as the main effect means with their corresponding Standard Error (SE). Within the same column, means annotated with identical letters do not exhibit statistically significant differences as determined by the Least Significant Difference (LSD) test. Significance levels are denoted by * (P ≤ 0.05) and ** (P ≤ 0.01) based on the F-test; ‘ns’ indicates a non-significant result. Chemical Fertilizer Application (CF), was tested at three levels, 100% (CF100), 75% (CF75), and 50% (CF50) of the fertilizer requirement as determined by soil testing. This was coupled with foliar applications of a balanced 20-20-20 NPK fertilizer enriched with micronutrients. Biological Fertilizer Application (BF), also comprised three levels: BF0 (control), where trees received only soil-applied organic fertilizer without biological agents; BF1, which included a soil application of an organic fertilizer mix, mycorrhizal fungi, and the beneficial bacteria Bacillus subtilis and Pseudomonas fluorescens, supplemented with fulvic acid and amino acids; and BF1+BFF, where trees were treated with both soil and foliar applications of the aforementioned bacterial species, fulvic acid, and amino acids.

Bold values: Olive leaf nutrients sufficient range according to (Kailis and Harris, 2007; Fernández-Escobar et al., 2009; Ali, 2023). Olive leaf tissue analysis from sub terminal leaflets of current year’s non-fruit bearing growth.

The application of organic matter to soil, in conjunction with biofertilizers, has been shown to significantly influence the leaf nutrient concentrations N, P, K, S, Fe, Mn, Zn, and Cu (**Tables 4**, **5**). Specifically, the BF1+BFF treatment, which involves both soil application and foliar spray of biofertilizers, led to the most substantial increase in the concentrations of leaf N, P, K, S, Fe, Mn, Zn, and Cu. The increments were 34.94%, 77.78%, 46.67%, 53.85%, 55.14%, 45.02%, 90.64%, and 39.4%, respectively. Notably, this treatment showed no significant difference from the BF1 treatment (soil application of biofertilizers alone) in terms of elevating the leaf nutrient concentrations of N, P, K, and S. However, the BF1 treatment was ranked in the second statistical group for the enhancement of Fe, Mn, Zn, and Cu concentrations. This suggests that the foliar application of biofertilizers has a marked impact on increasing these specific leaf nutrients in the Zard olive cultivar.

The interaction effect of the concurrent application of chemical fertilizers (CF) and biofertilizers (BF) on leaf nutrient content was found to be significant. Notably, the treatments CF100+BF1+BFF, CF100+BF1, and CF75+BF1+BFF demonstrated the most pronounced enhancement in the foliar concentrations of N, P, K, Ca, Mg, and S, aligning them within the same statistical group as indicated in **Table 4**. The combination of CF100 with BF1+BFF showed the highest increase in N content by 31.37%, P content by 58.33%, K content by 38.89%, S content by 43.75%, Ca content by 34.62%, Mg content by 17.07%, Fe content by 57.94%, Mn content by 40.23%, Zn content by 32.87%, and Cu content by 20.28% compared to CF100+BF0 (control). Specifically, the CF75+BF1 treatment was distinguished as the leading group for elevating the foliar levels of P, K, and Mg. Furthermore, the treatments CF100+BF1+BFF and CF75+BF1+BFF were responsible for the highest increments in the foliar concentrations of Fe, Mn, Zn, Cu, and B in the Zard olive cultivar. With the exception of Fe, these treatments were categorized within the same statistical group. Additionally, these treatments, in conjunction with CF50+BF1+BFF, were grouped together for their role in augmenting the foliar B concentration.

### Fruit characteristics of olive trees

The application of chemical fertilizers, significantly influenced the quantitative traits of olive fruits, such as fruit length, diameter, and both fresh and dry weights, as well as the average yield per tree, with the exception of fruit flesh thickness (**Table 6**). The most pronounced values for these traits were observed in the CF100 (control) and CF75 treatments, which were categorized within the same statistical group. A 50% reduction in the soil application of chemical fertilizers, as determined by soil testing (CF50), led to a significant decrease in the quantitative traits of the fruit, including reductions in fruit length (by 21.47%), fruit diameter (by 22.25%), both fresh and dry weights (by 9.73% and 10.87%, respectively), and the average yield per tree (by 33.19%) of the Zard olive cultivar, when compared to the control, thereby assigning it to the second statistical group.

**Table 6:** The simple and interactive effects of chemical and biological fertilizers on the fruit characteristics of olive trees (*Olea europaea* L., cv. ‘Zard’).

Treatment	Fruit length (mm)	Fruit diameter (mm)	Fruit flesh thickness (mm)	Fruit fresh weight (g)	Fruit dry weight (g)	Fruit yield (kg per tree)
I- Chemical fertilizers						
CF100 (control)	28.42 ± 2.53a	21.68 ± 2.14a	4.69 ± 0.42a	4.52 ± 0.21a	2.30 ± 0.12a	45.17 ± 3.26a
CF75	26.15 ± 2.48ab	19.72 ± 2.08ab	4.27 ± 0.39a	4.29 ± 0.20ab	2.14 ± 0.12ab	39.62 ± 2.64a
CF50	22.31 ± 2.71b	16.85 ± 1.46b	4.03 ± 0.27a	4.08 ± 0.19b	2.05 ± 0.10b	30.18 ± 2.09b
**F. test**	**	**	**ns**	**	**	**
II- Biological fertilizers						
BF0 (control)	21.23 ± 2.15b	14.63 ± 1.21c	3.74 ± 0.25c	3.52 ± 0.23c	1.67 ± 0.14c	31.36 ± 2.78c
BF1	26.12 ± 2.48a	19.81 ± 1.74b	4.31 ± 0.31b	4.22 ± 0.21b	2.15 ± 0.20b	38.53 ± 3.06b
BF1+BFF	29.53 ± 2.73a	23.82 ± 2.15a	4.94 ± 0.32a	5.14 ± 0.32a	2.66 ± 0.21a	45.09 ± 3.32a
**F. test**	**	**	**	**	**	**
Interaction						
CF100+BF0 (Control)	24.15 ± 2.15bc	17.26 ± 1.43cd	4.13 ± 0.30c	3.87 ± 0.19c	1.85 ± 0.14c	39.13 ± 2.95b
CF100+BF1	29.30 ± 2.48a	22.94 ± 2.02ab	4.69 ± 0.31ab	4.31 ± 0.24b	2.17 ± 0.21bc	46.70 ± 3.54a
CF100+ BF1+BFF	31.80 ± 3.02a	24.84 ± 2.18a	5.26 ± 0.39a	5.37 ± 0.36a	2.88 ± 0.22a	49.69 ± 3.61a
CF75+BF0	21.46 ± 2.11cd	14.25 ± 1.23e	3.83 ± 0.25c	3.49 ± 0.18d	1.68 ± 0.13d	33.40 ± 2.81c
CF75+BF1	26.91 ± 2.35ab	20.06 ± 1.61bc	4.07 ± 0.28c	4.29 ± 0.23b	2.12 ± 0.19b	39.15 ± 3.02b
CF75+ BF1+BFF	30.08 ± 2.81a	24.85 ± 2.09a	4.91 ± 0.34a	5.08 ± 0.29a	2.61 ± 0.23a	46.31 ± 3.57a
CF50+BF0	18.07 ± 1.60d	12.37 ± 1.13e	3.27 ± 0.20d	3.21 ± 0.16d	1.49 ± 0.12d	21.54 ± 2.38d
CF50+BF1	22.14 ± 2.01c	16.43 ± 1.34d	4.17 ± 0.27bc	4.05 ± 0.22bc	2.15 ± 0.20bc	29.75 ± 2.43c
CF50+ BF1+BFF	26.71 ± 2.27ab	21.76 ± 1.88ab	4.65 ± 0.29ab	4.97 ± 0.28a	2.50 ± 0.22ab	39.26 ± 2.91b
**F. test**	**	**	**	**	**	**

The data are presented as the main effect means with their corresponding Standard Error (SE). Within the same column, means annotated with identical letters do not exhibit statistically significant differences as determined by the Least Significant Difference (LSD) test. Bold text: Significance levels are denoted by * (P ≤ 0.05) and ** (P ≤ 0.01) based on the F-test; ‘ns’ indicates a non-significant result. Chemical Fertilizer Application (CF), was tested at three levels, 100% (CF100), 75% (CF75), and 50% (CF50) of the fertilizer requirement as determined by soil testing. This was coupled with foliar applications of a balanced 20-20-20 NPK fertilizer enriched with micronutrients. Biological Fertilizer Application (BF), also comprised three levels: BF0 (control), where trees received only soil-applied organic fertilizer without biological agents; BF1, which included a soil application of an organic fertilizer mix, mycorrhizal fungi, and the beneficial bacteria Bacillus subtilis and Pseudomonas fluorescens, supplemented with fulvic acid and amino acids; and BF1+BFF, where trees were treated with both soil and foliar applications of the aforementioned bacterial species, fulvic acid, and amino acids.

The soil and foliar application of biological fertilizer, in conjunction with organic matter, markedly influenced all quantitative traits of the Zard cultivar olive fruit. The treatment involving soil application of biological fertilizer and organic matter, coupled with foliar application of biological fertilizer BF1+BFF, exhibited the most substantial enhancement in the quantitative traits of the Zard cultivar olive fruit, in comparison to the control (absence of biological fertilizer application). The application of BF1+BFF treatment was observed to enhance various fruit growth parameters in comparison with the BF0 (control). The results indicated a significant increase in fruit length by 39.11%, fruit diameter by 62.80%, fruit flesh thickness by 32.09%, fruit fresh weight by 46.02%, fruit dry weight by 59.28%, and fruit yield by 43.72%, as presented in **Table 6**.

The interactive effects of chemical and biological fertilizers were found to be significant on the quantitative traits of the Zard cultivar olive fruit, as detailed in **Table 6**. The data presented in **Table 6** suggest that the most pronounced measurements of quantitative traits, including fruit length, diameter, flesh thickness, and overall fruit weight, were associated with the treatments CF100+BF1+BFF, CF75+BF1+BFF, CF100+BF1, and CF50+BF1+BFF. Additionally, the highest values for both fresh and dry fruit weights were observed in treatments CF100+BF1+BFF, CF75+BF1+BFF, and CF50+BF1+BFF. This highlights the advantageous impact of the foliar application of biological fertilizers in combination with chemical fertilizers on the enhancement of the Zard cultivar olive fruit’s weight. Moreover, the most substantial fruit yields correlated with treatments CF100+BF1+BFF, CF100+BF1, and CF75+BF1+BFF, which were categorized within the same statistical group. Following these, treatments CF50+BF1+BFF, CF75+BF1, and CF100+BF0 (Control) were classified in the subsequent statistical category. The CF100+BF1+BFF treatment resulted in increases in fruit length by 31.14%, fruit diameter by 41.61%, fruit flesh thickness by 30.48%, fruit fresh weight by 38.76%, fruit dry weight by 55.68%, and yield per tree by 27.00% compared to the control (CF100+BF0). These results demonstrate that the combined soil and foliar application of chemical fertilizers with biological fertilizers (BF1+BFF) led to the highest increases in all measured fruit traits, underscoring the efficacy of this treatment in promoting olive fruit growth and yield.

There was no significant change in water content, oil content (fresh and dry weight), or free acidity across the chemical fertilizer treatments. The impact of the chemical fertilizers on the peroxide value of olive oil, was found to be statistically significant (**Table 7**). The treatments CF75 and CF100 (control) yielded the lowest observed values for these characteristics, grouping them within the same statistical category. Peroxide value showed a significant increase when the fertilizer was reduced to 50% (CF50) of the control amount (CF100), with an increase of 11.73%.

**Table 7:** The simple and interactive effects of chemical and biological fertilizers on the olive oil quality of ‘zard’ olive trees (*Olea europaea* L., cv. ‘Zard’).

Treatment					
I- Chemical fertilizers	Water Content (% of Fresh Weight)	Oil Content (% of Fresh Weight)	Oil Content (% of Dry Weight)	Free Acidity (%)	Peroxide Value (Meq O_2_ kg/Oil)
CF100 (control)	51.70 ± 3.10a	23.68 ± 1.02a	45.98 ± 2.50a	0.58 ± 0.03a	3.92 ± 0.19b
CF75	50.10 ± 3.03a	23.16 ± 0.97a	45.19 ± 2.41a	0.54 ± 0.02a	3.67 ± 0.17b
CF50	48.86 ± 2.30a	22.29 ± 0.93a	44.41 ± 2.22a	0.54 ± 0.02a	4.38 ± 0.24a
**F. test**	ns	**ns**	**ns**	**ns**	**
II- Biological fertilizers					
BF0 (control)	47.31 ± 2.22b	21.57 ± 0.86b	42.66 ± 2.00b	0.64 ± 0.03a	4.74 ± 0.27a
BF1	50.75 ± 3.15ab	22.93 ± 0.89a	45.48 ± 2.55ab	0.56 ± 0.03b	3.99 ± 0.20b
BF1+BFF	52.60 ± 3.02a	24.64 ± 1.16a	47.44 ± 2.68a	0.46 ± 0.02c	3.24 ± 0.12c
**F. test**	**	**	**	**	**
Interaction					
CF100+BF0 (Control)	48.92 ± 2.33bc	22.56 ± 0.87cd	42.63 ± 2.02bc	0.67 ± 0.03a	4.85 ± 0.29a
CF100+BF1	51.27 ± 3.13abc	23.19 ± 0.92bc	46.72 ± 2.70abc	0.59 ± 0.03b	3.85 ± 0.19c
CF100+ BF1+BFF	54.92 ± 3.00a	25.30 ± 1.13a	48.60 ± 2.81a	0.48 ± 0.02d	3.06 ± 0.11e
CF75+BF0	47.02 ± 2.13bc	21.74 ± 0.77de	43.05 ± 2.11bc	0.64 ± 0.03a	4.06 ± 0.21bc
CF75+BF1	51.13 ± 3.06abc	22.98 ± 0.80bc	45.39 ± 2.54abc	0.57 ± 0.03bc	3.81 ± 0.18cd
CF75+ BF1+BFF	52.16 ± 3.04ab	24.76 ± 1.18ab	47.13 ± 2.61ab	0.42 ± 0.02e	3.15 ± 0.12e
CF50+BF0	46.00 ± 2.45c	20.40 ± 0.74e	42.30 ± 2.01c	0.61 ± 0.03b	5.32 ± 0.33a
CF50+BF1	49.85 ± 2.29abc	22.61 ± 0.96cd	44.33 ± 2.20abc	0.53 ± 0.02c	4.31 ± 0.23b
CF50+ BF1+BFF	50.72 ± 2.85abc	23.85 ± 0.99abc	46.60 ± 2.66abc	0.49 ± 0.02d	3.52 ± 0.15d
**F. test**	**	**	*	**	**

The data are presented as the main effect means with their corresponding Standard Error (SE). Within the same column, means annotated with identical letters do not exhibit statistically significant differences as determined by the Least Significant Difference (LSD) test. Bold text: Significance levels are denoted by * (P ≤ 0.05) and ** (P ≤ 0.01) based on the F-test; ‘ns’ indicates a non-significant result. Chemical Fertilizer Application (CF), was tested at three levels, 100% (CF100), 75% (CF75), and 50% (CF50) of the fertilizer requirement as determined by soil testing. This was coupled with foliar applications of a balanced 20-20-20 NPK fertilizer enriched with micronutrients. Biological Fertilizer Application (BF), also comprised three levels: BF0 (control), where trees received only soil-applied organic fertilizer without biological agents; BF1, which included a soil application of an organic fertilizer mix, mycorrhizal fungi, and the beneficial bacteria Bacillus subtilis and Pseudomonas fluorescens, supplemented with fulvic acid and amino acids; and BF1+BFF, where trees were treated with both soil and foliar applications of the aforementioned bacterial species, fulvic acid, and amino acids.

Soil biofertilizer application, in conjunction with organic matter and foliar biofertilizer treatments, significantly altered all quality parameters of the ‘Zard’ olive cultivar. The most substantial increases in water content (by 11.26% and 7.21%), oil content in the fresh fruit weight (by 14.24% and 6.34%), and in the dry fruit weight (by 11.21% and 6.62%) were observed in treatments involving soil application of biofertilizer with organic matter and foliar biofertilizer BF1+BFF, and soil application of biofertilizer with organic matter BF1, respectively, when compared to the control. Furthermore, the lowest levels of free acidity (28.12%) and peroxide value (31.65%) in olive oil, relative to the control, were associated with the combined soil and foliar application of biofertilizer BF1+BFF, followed by the soil application of biofertilizer with organic matter BF1, which demonstrated reductions in free acidity (12.5%) and peroxide value (15.8%) within the second statistical grouping.

The interaction effects of chemical and biological fertilizers on the quality attributes of ‘Zard’ olive oil was found to be statistically significant, as detailed in **Table 7**. In the Zard cultivar of olive fruit, the maximal water content, oil content on a fresh weight basis, and oil content on a dry weight basis were recorded in the treatment group CF100+BF1+BFF. These values showed an increase of 12.31%, 12.14%, and 14.01%, respectively, in comparison to the control group CF100+BF0. The data in **Table 7** reveal that the treatments CF100+BF1+BFF, CF75+BF1+BFF, CF100+BF1, and CF50+BF1+BFF, all within the same statistical category, exhibited the highest increases in fruit water content in the fresh fruit weight, and oil content in both the fresh and dry fruit weights, compared to the control. Conversely, the lowest levels of free acidity and peroxide value in the ‘Zard’ olive oil were recorded for the treatments CF100+ BF1+BFF (by 28.42% and 36.91%) and CF75+ BF1+BFF (by 37.31% and 35.11%), with the treatment CF50+BF1+BFF following closely. These findings underscore the beneficial influence of foliar biofertilizer application in tandem with chemical fertilizers in diminishing the free acidity and peroxide values in ‘Zard’ olive oil.

The application of chemical fertilizers has been observed to significantly influence the K_268_ and ΔK as the oxidative properties of Zard olive oil cultivar (**Table 8**). The K_268_ value decreased significantly when the chemical fertilizer was reduced to 75% (CF75) and 50% (CF50) of the control amount (CF100), with changes of -9.26% and -18.52%, respectively. In a similar trend, these treatments markedly lowered the oxidative parameter ΔK for the Zard olive oil cultivar by 94.11% and 235.29%, respectively, when contrasted with the control treatment CF100.

**Table 8:** The simple and interactive effects of chemical and biological fertilizers on oxidation parameters (K_270_, K_232_, K_262_, K_268_, K_274_ and ΔK) in ‘Zard’ olive oil (*Olea europaea* L., cv. ‘Zard’).

Treatment	K_270_	K_232_	K_262_	K_268_	K_274_	ΔK
I- Chemical fertilizers						
CF100 (control)	0.183 ± 0.008a	1.744 ± 0.07a	0.223 ± 0.009a	0.216 ± 0.010a	0.174 ± 0.007a	0.017 ± 0.0010a
CF75	0.182 ± 0.007a	1.650 ± 0.06a	0.221 ± 0.009a	0.196 ± 0.008b	0.168 ± 0.006a	0.001 ± 0.0003b
CF50	0.190 ± 0.009a	1.743 ± 0.07a	0.216 ± 0.008a	0.176 ± 0.007c	0.181 ± 0.007a	-0.023 ± 0.0012c
**F. test**	ns	**ns**	**ns**	**	**ns**	**
II- Biological fertilizers						
BF0 (control)	0.198 ± 0.010a	1.847 ± 0.07a	0.229 ± 0.010a	0.207 ± 0.008a	0.184 ± 0.007a	0.0001 ± 0.0000b
BF1	0.187 ± 0.008a	1.703 ± 0.07b	0.220 ± 0.008a	0.200 ± 0.009a	0.176 ± 0.006a	0.0020 ± 0.0004a
BF1+BFF	0.169 ± 0.006b	1.588 ± 0.05c	0.211 ± 0.006b	0.181 ± 0.008b	0.164 ± 0.005b	-0.0060 ± 0.0008c
**F. test**	**	**	**	**	**	**ns**
Interaction						
CF100+BF0 (Control)	0.192 ± 0.009ab	1.852 ± 0.08ab	0.229 ± 0.010ab	0.228 ± 0.011a	0.181 ± 0.007ab	0.0230 ± 0.0011a
CF100+BF1	0.186 ± 0.009bcd	1.761 ± 0.07b	0.224 ± 0.008ab	0.221 ± 0.009a	0.180 ± 0.007ab	0.0190 ± 0.0008a
CF100+ BF1+BFF	0.171 ± 0.007de	1.620 ± 0.06e	0.217 ± 0.007bcd	0.198 ± 0.008bc	0.162 ± 0.005c	0.0085 ± 0.0009b
CF75+BF0	0.197 ± 0.010ab	1.738 ± 0.06c	0.234 ± 0.009a	0.214 ± 0.008ab	0.179 ± 0.006ab	0.0075 ± 0.0008b
CF75+BF1	0.184 ± 0.008cd	1.675 ± 0.06d	0.221 ± 0.007abc	0.193 ± 0.008c	0.166 ± 0.005c	-0.0005 ± 0.0000c
CF75+ BF1+BFF	0.164 ± 0.006e	1.538 ± 0.06g	0.207 ± 0.006d	0.181 ± 0.007d	0.160 ± 0.005c	-0.0025 ± 0.0003d
CF50+BF0	0.206 ± 0.010a	1.952 ± 0.08a	0.224 ± 0.006ab	0.178 ± 0.006d	0.191 ± 0.008a	-0.0295 ± 0.0015g
CF50+BF1	0.191 ± 0.008abc	1.673 ± 0.06d	0.214 ± 0.006bcd	0.185 ± 0.007cd	0.182 ± 0.007ab	-0.0130 ± 0.0006e
CF50+ BF1+BFF	0.173 ± 0.007de	1.605 ± 0.05f	0.209 ± 0.006cd	0.164 ± 0.006e	0.169 ± 0.006bc	-0.0250 ± 0.0012f
**F. test**	*	**	**	**	**	**
**Extra virgin classification**	<0.22	<2.5		<0.22		<0.01

The data are presented as the main effect means with their corresponding Standard Error (SE). Within the same column, means annotated with identical letters do not exhibit statistically significant differences as determined by the Least Significant Difference (LSD) test. Bold text: Significance levels are denoted by * (P ≤ 0.05) and ** (P ≤ 0.01) based on the F-test; ‘ns’ indicates a non-significant result. Chemical Fertilizer Application (CF), was tested at three levels, 100% (CF100), 75% (CF75), and 50% (CF50) of the fertilizer requirement as determined by soil testing. This was coupled with foliar applications of a balanced 20-20-20 NPK fertilizer enriched with micronutrients. Biological Fertilizer Application (BF), also comprised three levels: BF0 (control), where trees received only soil-applied organic fertilizer without biological agents; BF1, which included a soil application of an organic fertilizer mix, mycorrhizal fungi, and the beneficial bacteria Bacillus subtilis and Pseudomonas fluorescens, supplemented with fulvic acid and amino acids; and BF1+BFF, where trees were treated with both soil and foliar applications of the aforementioned bacterial species, fulvic acid, and amino acids.

The application of biological fertilizers, in conjunction with organic matter and foliar biofertilizer application, significantly altered all oxidative parameters of Zard cultivar olive oil, with the exception of the oxidative characteristic ΔK, at a 1% statistical significance level. The K_270_ value showed a significant decrease with the combined soil and foliar application of biofertilizer BF1+BFF (BF1+BFF), with a change of -14.65% compared to the control (BF0). K_232_, K_262_, K_268_, and K_274_ values also decreased significantly with the application of BF1+BFF, with changes of -14.01%, -7.86%, -12.56%, and -10.87%, respectively.

The interaction between chemical and biological fertilizers significantly affects the oxidative parameters of Zard cultivar olive oil, as illustrated in **Table 8**. The treatments combining 75% chemical fertilizer with biofertilizer (CF75+BF1+BFF), 50% chemical fertilizer with biofertilizer (CF50+BF1+BFF), and 100% chemical fertilizer with biofertilizer (CF100+BF1+BFF) were associated with the lowest levels of oxidative characteristics, namely K_270_, K_232_, K_262_, and K_274_, when compared to the control treatment (CF100+BF0). Specifically, the full chemical fertilizer combined with both soil and foliar application of biofertilizer (CF100+BF1+BFF) resulted in a significant reduction in K_232_ by -12.54% relative to the control. Moreover, the 75% chemical fertilizer blend with biofertilizer (CF75+BF1+BFF) exhibited the most substantial decrease in K_232_, at -16.95%. Notably, the 50% chemical fertilizer mix with biofertilizer (CF50+BF1+BFF) led to the most pronounced reduction in ΔK, at -208.70%. These findings underscore the positive impact of foliar biofertilizer application in tandem with chemical fertilizers on reducing the oxidative characteristics of Zard cultivar olive oil. Additionally, the treatments CF50+BF1+BFF and CF50+BF0 corresponded to the lowest oxidative characteristic values for K_268_ and ΔK, respectively. This emphasizes the beneficial effects of foliar biofertilizer application, both independently and in combination with chemical fertilizers, on diminishing the free acidity and peroxide value of Zard cultivar olive oil.

The reduction in chemical fertilizer application from 100% (CF100) to 50% (CF50) led to a decrease in palmitic acid (C16:0) by approximately 11.7% (**Table 9**). This suggests that a lower chemical fertilizer input can effectively reduce the palmitic acid content in olive oil, potentially contributing to a healthier oil profile.

The use of biological fertilizers (BF1+BFF) resulted in a significant decrease in Palmitic Acid (C16:0) by about 27.2% compared to the control (BF0) (**Table 9**). This indicates that biological fertilizers can play a crucial role in altering the fatty acid composition towards a healthier profile.

**Table 9:** Simple and interactive effects of chemical and biological fertilizers on major fatty acid concentrations (% of total fatty acids) in ‘Zard’ olive oil (*Olea europaea* L.).

Treatment	Palmitic (C16:0)	Stearic (C18:0)	Arachidic (C20:0)	Palmitoleic (C16:1c9)	Oleic (C18:1c9)	Eicosenoic (C20:1c11)	Linoleic (C18:2c9c12)	a-linolenic C18:3c5c9c12)
I- Chemical fertilizers								
CF100 (control)	13.01 ± 0.70a	2.72 ± 0.13a	0.464 ± 0.015a	0.816 ± 0.031a	73.96 ± 3.20a	0.258 ± 0.013a	6.675 ± 0.27a	0.816 ± 0.032a
CF75	12.30 ± 0.63a	2.57 ± 0.12a	0.441 ± 0.014a	0.812 ± 0.030a	77.62 ± 3.51a	0.275 ± 0.014a	6.202 ± 0.26a	0.735 ± 0.024b
CF50	11.49 ± 0.55b	2.61 ± 0.13a	0.446 ± 0.015a	0.806 ± 0.030a	74.00 ± 3.35a	0.262 ± 0.013a	6.457 ± 0.27a	0.731 ± 0.023b
**F. test**	**	**ns**	**ns**	**ns**	**ns**	**ns**	**ns**	**
II- Biological fertilizers								
BF0 (control)	14.28 ± 0.73a	2.87 ± 0.14a	0.466 ± 0.017a	0.882 ± 0.034a	70.77 ± 3.14b	0.243 ± 0.012a	7.204 ± 0.28a	0.912 ± 0.034a
BF1	12.05 ± 0.61b	2.57 ± 0.12b	0.450 ± 0.015a	0.810 ± 0.031b	75.05 ± 3.36ab	0.265 ± 0.013a	6.650 ± 0.27b	0.734 ± 0.023b
BF1+BFF	10.46 ± 0.55c	2.46 ± 0.12b	0.436 ± 0.014a	0.741 ± 0.026c	79.77 ± 3.60a	0.286 ± 0.014a	5.480 ± 0.23c	0.636 ± 0.021c
**F. test**	**	**	**ns**	**	**	**ns**	**	**
Interaction								
CF100+BF0 (Control)	15.37 ± 0.84a	2.98 ± 0.14a	0.498 ± 0.017a	0.917 ± 0.038a	68.35 ± 2.86c	0.225 ± 0.011d	7.931 ± 0.29a	0.995 ± 0.037a
CF100+BF1	12.15 ± 0.62bc	2.71 ± 0.13ab	0.461 ± 0.016ab	0.814 ± 0.031b	74.12 ± 3.11bc	0.267 ± 0.013abc	6.843 ± 0.26b	0.739 ± 0.024c
CF100+ BF1+BFF	11.50 ± 0.55c	2.47 ± 0.12bc	0.433 ± 0.014b	0.716 ± 0.025e	79.40 ± 3.55ab	0.281 ± 0.013a	5.251 ± 0.22d	0.713 ± 0.021c
CF75+BF0	14.78 ± 0.78a	2.82 ± 0.13a	0.447 ± 0.016b	0.865 ± 0.037ab	74.35 ± 3.22bc	0.261 ± 0.012bc	6.930 ± 0.27b	0.892 ± 0.032b
CF75+BF1	11.91 ± 0.59bc	2.48 ± 0.12bc	0.442 ± 0.015b	0.813 ± 0.031bc	77.12 ± 3.61ab	0.269 ± 0.012ab	6.490 ± 0.25bc	0.731 ± 0.023c
CF75+ BF1+BFF	10.21 ± 0.52d	2.41 ± 0.11c	0.435 ± 0.014b	0.759 ± 0.026c	81.40 ± 3.74a	0.294 ± 0.014a	5.187 ± 0.22d	0.582 ± 0.019d
CF50+BF0	12.70 ± 0.67b	2.82 ± 0.13a	0.453 ± 0.016b	0.864 ± 0.036ab	69.60 ± 2.75c	0.243 ± 0.011cd	6.750 ± 0.26b	0.849 ± 0.028b
CF50+BF1	12.10 ± 0.61bc	2.51 ± 0.12bc	0.446 ± 0.015b	0.804 ± 0.030bcd	73.90 ± 3.03bc	0.259 ± 0.012bc	6.618 ± 0.26b	0.733 ± 0.023c
CF50+ BF1+BFF	9.67 ± 0.47d	2.49 ± 0.12bc	0.439 ± 0.014b	0.749 ± 0.025de	78.50 ± 3.64ab	0.284 ± 0.013ab	6.003 ± 0.24c	0.612 ± 0.020d
**F. test**	**	**	*	**	**	**	**	**

The data are presented as the main effect means with their corresponding Standard Error (SE). Within the same column, means annotated with identical letters do not exhibit statistically significant differences as determined by the Least Significant Difference (LSD) test. Bold text: Significance levels are denoted by * (P ≤ 0.05) and ** (P ≤ 0.01) based on the F-test; ‘ns’ indicates a non-significant result. Chemical Fertilizer Application (CF), was tested at three levels, 100% (CF100), 75% (CF75), and 50% (CF50) of the fertilizer requirement as determined by soil testing. This was coupled with foliar applications of a balanced 20-20-20 NPK fertilizer enriched with micronutrients. Biological Fertilizer Application (BF), also comprised three levels: BF0 (control), where trees received only soil-applied organic fertilizer without biological agents; BF1, which included a soil application of an organic fertilizer mix, mycorrhizal fungi, and the beneficial bacteria Bacillus subtilis and Pseudomonas fluorescens, supplemented with fulvic acid and amino acids; and BF1+BFF, where trees were treated with both soil and foliar applications of the aforementioned bacterial species, fulvic acid, and amino acids.

The combined treatment of 75% chemical fertilizer and biological fertilizers (CF75+BF1+BFF) resulted in the most significant decrease in stearic acid (C18:0) by 19.1%, arachidic acid (C20:0) by 13.1%, palmitoleic acid (C16:1c9) by 21.9%, and linoleic acid (C18:2c9c12) by 33.8% (**Table 9**). The interaction effect of this combined treatment showed the most pronounced effects on the fatty acid profile. Specifically, this treatment led to the highest increase in eicosenoic acid (C20:1c11) by 30.7% and oleic acid (C18:1c9) by 19.0%, as well as the largest decrease in α-Linolenic Acid (C18:3c5c9c12) by 41.6% compared to the control (CF100+BF0). These changes are particularly noteworthy as they suggest that a synergistic effect of both fertilizer types can optimize the fatty acid composition for improved oil quality.

Most leaf nutrients (N, P, K, Ca, Mg, S, Fe, Mn, Zn, Cu, B) show strong positive correlations with various fruit characteristics such as fruit length, diameter, flesh thickness, fresh weight, dry weight, and yield. This suggests that higher nutrient content is associated with larger and heavier fruits (**Table 10**). There is a very strong positive correlation between all nutrients and fruit length, especially with Phosphorus (P) at 0.987**. Both fresh and dry weights of fruits have very strong positive correlations with all nutrients, particularly with Sulfur (S) for fresh weight (0.986**) and Phosphorus (P) for dry weight (0.974**). High positive correlations suggest that nutrients are important for the water content in fruits. Both in fresh and dry weight, there is a strong positive correlation with all nutrients, highlighting the importance of nutrients for oil content in fruits. The oil quality parameters such as free acidity and peroxide value show a strong negative correlation with all nutrients, indicating that higher nutrient content may be associated with lower acidity and peroxide value, which are desirable traits. K_270_, K_232_, K_274_ parameters, which are related to oil quality, show strong negative correlations with nutrients, suggesting that higher nutrient content might improve oil quality. The correlations vary, with some fatty acids like Oleic acid showing a positive correlation with nutrients, while others like Linoleic acid show a negative correlation. Certain nutrients like Boron (B) show a unique pattern of correlation, with a strong positive correlation with fruit oil content in fresh weight (0.791**) but a strong negative correlation with free acidity (-0.741**).

**Table 10:** The correlation between leaf nutrient content and different fruit characteristics.

Fruit characteristics	N	P	K	Ca	Mg	S	Fe	Mn	Zn	Cu	B
Fruit length (mm)	0.912^**^	0.987^**^	0.949^**^	0.959^**^	0.806^**^	0.977^**^	0.948^**^	0.977^**^	0.958^**^	0.812^**^	0.653^*^
Fruit diameter (mm)	0.833^**^	0.953^**^	0.893^**^	0.921^**^	0.722^**^	0.934^**^	0.892^**^	0.940^**^	0.935^**^	0.775^**^	0.699^*^
Fruit flesh thickness (mm)	0.693^*^	0.859^**^	0.759^**^	0.841^**^	0.559^*^	0.814^**^	0.827^**^	0.881^**^	0.871^**^	0.748^**^	0.825^**^
Fruit fresh weight (g)	0.678^*^	0.833^**^	0.740^**^	0.846^**^	0.543^*^	0.789^**^	0.821^**^	0.866^**^	0.834^**^	0.688^**^	0.802^**^
Fruit dry weight (g)	0.962^**^	0.974^**^	0.960^**^	0.938^**^	0.896^**^	0.986^**^	0.944^**^	0.977^**^	0.977^**^	0.861^**^	0.606^*^
Fruit yield (kg tree^-1^)	0.812^**^	0.898^**^	0.831^**^	0.923^**^	0.713^**^	0.888^**^	0.888^**^	0.937^**^	0.923^**^	0.771^**^	0.762^**^
Water Content in fresh weight (%)	0.817^**^	0.919^**^	0.861^**^	0.940^**^	0.667^*^	0.889^**^	0.925^**^	0.926^**^	0.874^**^	0.702^**^	0.665^*^
Fruit oil content in fresh weight (%)	0.799^**^	0.906^**^	0.841^**^	0.885^**^	0.689^*^	0.877^**^	0.889^**^	0.941^**^	0.922^**^	0.794^**^	0.791^**^
Fruit oil content in dry weight (%)	0.733^**^	0.880^**^	0.805^**^	0.905^**^	0.607^*^	0.849^**^	0.843^**^	0.879^**^	0.827^**^	0.661^*^	0.709^**^
Free Acidity (%)	-0.334	-0.554^*^	-0.461 ^ns^	-0.537^*^	-0.209 ^ns^	-0.489 ^ns^	-0.497 ^ns^	-0.579^*^	-0.532^*^	-0.404 ^ns^	-0.741^**^
Peroxide Value (Meq O_2_ kg/Oil)	-0.743^**^	-0.843^**^	-0.816^**^	-0.800^**^	-0.715^**^	-0.812^**^	-0.790^**^	-0.867^**^	-0.810^**^	-0.686^*^	-0.685^*^
K_270_	-0.689^*^	-0.847^**^	-0.771^**^	-0.770^**^	-0.598^*^	-0.804^**^	-0.777^**^	-0.864^**^	-0.868^**^	-0.757^**^	-0.806^**^
K_232_	-0.524^*^	-0.660^*^	-0.631^*^	-0.586^*^	-0.545^*^	-0.606^*^	-0.586^*^	-0.692^*^	-0.650^*^	-0.492 ^ns^	-0.606^*^
K_262_	-0.110 ^ns^	-0.382 ^ns^	-0.254 ^ns^	-0.353 ^ns^	0.001 ^ns^	-0.305 ^ns^	-0.296 ^ns^	-0.380 ^ns^	-0.389 ^ns^	-0.228 ^ns^	-0.608^*^
K_268_	0.423 ^ns^	0.139 ^ns^	0.272 ^ns^	0.167 ^ns^	0.515 ^ns^	0.233 ^ns^	0.211 ^ns^	0.149 ^ns^	0.156 ^ns^	0.165 ^ns^	-0.341 ^ns^
K_274_	-0.724^**^	-0.842^**^	-0.809^**^	-0.722^**^	-0.641^*^	-0.789^**^	-0.788^**^	-0.850^**^	-0.815^**^	-0.761^**^	-0.713^**^
ΔK	0.734^**^	0.500^*^	0.616^*^	0.491 ^ns^	0.791^**^	0.577^*^	0.548^*^	0.514 ^ns^	0.515 ^ns^	0.470 ^ns^	-0.046 ^ns^
Palmitic (C16:0)	-0.155 ^ns^	-0.442 ^ns^	-0.315 ^ns^	-0.422 ^ns^	-0.045 ^ns^	-0.368 ^ns^	-0.331 ^ns^	-0.405 ^ns^	-0.391 ^ns^	-0.239 ^ns^	-0.563^*^
Stearic (C18:0)	-0.312 ^ns^	-0.526^*^	-0.467 ^ns^	-0.497 ^ns^	-0.238 ^ns^	-0.446 ^ns^	-0.459 ^ns^	-0.513 ^ns^	-0.430 ^ns^	-0.241 ^ns^	-0.489 ^ns^
Arachidic (C20:0)	-0.101 ^ns^	-0.273 ^ns^	-0.237 ^ns^	-0.282 ^ns^	-0.048 ^ns^	-0.205 ^ns^	-0.235 ^ns^	-0.286 ^ns^	-0.151 ^ns^	-0.087 ^ns^	-0.427 ^ns^
Palmitoleic (C16:1c9)	-0.435 ^ns^	-0.643^**^	-0.537^*^	-0.687^*^	-0.300 ^ns^	-0.583^*^	-0.618^*^	-0.665^*^	-0.591^*^	-0.455 ^ns^	-0.731^**^
Oleic (C18:1c9)	0.542^*^	0.699^**^	0.662^*^	0.629^*^	0.500 ^ns^	0.644^*^	0.622^*^	0.711^**^	0.644^*^	0.541^*^	0.667^*^
Eicosenoic (C20:1c11)	0.459 ^ns^	0.633^*^	0.587^*^	0.588^*^	0.429 ^ns^	0.584^**^	0.538^*^	0.636^*^	0.571^*^	0.450 ^ns^	0.628^*^
Linoleic (C18:2c9c12)	-0.399 ^ns^	-0.586^*^	-0.510 ^ns^	-0.585^*^	-0.239 ^ns^	-0.528^*^	-0.561^*^	-0.617^*^	-0.525^*^	-0.474 ^ns^	-0.779^**^
a-linolenic C18:3c5c9c12)	-0.268 ^ns^	-0.513 ^ns^	-0.428 ^ns^	-0.479 ^ns^	-0.210 ^ns^	-0.448 ^ns^	-0.394 ^ns^	-0.486 ^ns^	-0.451 ^ns^	-0.267 ^ns^	-0.541^*^

*, ** significant in 5 and 1% statistical levels respectively ^ns^ not significant.

### Principal component analysis

The Principal Component Analysis (PCA) presented in **Table 11** for the olive (*Olea europaea* cv. ‘Zard’) reveals several important coefficients across the three main components (PC1, PC2, and PC3). These components represent the relationships between different variables related to leaf nutrient concentration, fruit characteristics, and oil quality characteristics. The coefficients indicate the strength and direction of the relationship between the variables and the principal components. Positive coefficients suggest a direct relationship, while negative coefficients imply an inverse relationship. The analysis yielded three principal components with eigenvalues above 1, and the accumulated variance accounted for 93.5% (**Table 11**). PC1 explains a substantial 70.4% of the variance. The dominant traits in this component are related to fruit characteristics, specifically, specifically, fruit flesh thickness (0.193), fruit fresh weight (0.191) and fruit oil content in fresh weight (0.193). PC2, accounting for 19.8% of the explained variance, is primarily associated with oil quality characteristics, particularly, K268 (0.357) and ΔK (0.355). 2. PC3, contributing 3.3% of the variance, highlights specific traits such as leaf B (0.436), Cu (0.284) concentration and Arachidic acid (C20:0) concentration (0.347). These loadings in PC3 suggest a unique variance explained by these components. Overall, the PCA provides a comprehensive understanding of the interrelationships among various traits of the ‘Zard’ olive cultivar. Notably, the first component (PC1) plays the most influential role in explaining the observed variability.

**Table 11:** Principal component analysis and coefficients of variables in 3 main components for determined characteristics of olive (*Olea europaea* cv. ‘Zard’).

Variable	PC1	PC2	PC3
Leaf nutrient concentration			
N	**0.158**	**0.218**	-0.103
P	**0.182**	0.129	0.011
K	**0.171**	0.164	-0.147
Ca	**0.176**	0.124	0.023
Mg	**0.133**	**0.23**	-0.32
S	**0.176**	0.16	-0.005
Fe	**0.174**	0.15	0.054
Mn	**0.185**	0.13	0.035
Zn	**0.176**	0.146	0.165
Cu	**0.151**	0.156	0.284
B	**0.152**	-0.048	0.436
Fruit characteristics			
Fruit length (mm)	**0.189**	0.1	0.008
Fruit diameter (mm)	**0.192**	0.047	0.065
Fruit flesh thickness (mm)	**0.193**	-0.031	0.16
Fruit fresh weight (g)	**0.191**	-0.04	0.124
Fruit dry weight (g)	**0.175**	0.171	0
Fruit yield (kg tree^-1^)	**0.188**	0.057	0.108
Water Content in fresh weight (%)	**0.187**	0.043	0.046
Fruit oil content in fresh weight (%)	**0.193**	0.029	0.1
Fruit oil content in dry weight (%)	**0.192**	-0.025	-0.021
Oil quality characteristics			
Free Acidity (%)	**-0.161**	**0.202**	-0.071
Peroxide Value (meq O_2_ kg/Oil)	**-0.189**	0.02	0.217
K_270_	**-0.191**	0.036	-0.101
K_232_	**-0.17**	0.102	0.213
K_262_	**-0.129**	**0.239**	-0.222
K_268_	**-0.038**	**0.357**	-0.158
K_274_	**-0.182**	0.016	0.076
ΔK	**0.041**	**0.355**	-0.145
Palmitic (C16:0)	**-0.136**	**0.245**	-0.069
Stearic (C18:0)	**-0.152**	**0.207**	0.188
Arachidic (C20:0)	**-0.107**	**0.252**	0.347
Palmitoleic (C16:1c9)	**-0.171**	0.163	0.008
Oleic (C18:1c9)	**0.177**	-0.125	-0.228
Eicosenoic (C20:1c11)	**0.167**	-0.156	-0.248
Linoleic (C18:2c9c12)	**-0.16**	0.182	0.019
a-linolenic C18:3c5c9c12)	**-0.15**	**0.22**	0.096
Eigenvalues	25.344	7.133	1.191
Explained variance (%)	0.704	0.198	0.033
Accumulated variance (%)	0.704	0.902	0.935
Selection criterion (SC)	0.099	0.187	0.458

Values in boldface are dominant in the PC loadings by setting the level of significance defined according to the selection criterion (SC=0.5/√ Eigenvalues). According to the SC, the boldface coefficients in the table are considered dominant in the PC loadings. This means that the traits associated with these coefficients are significantly related to the respective principal components.

**Figure 1:**
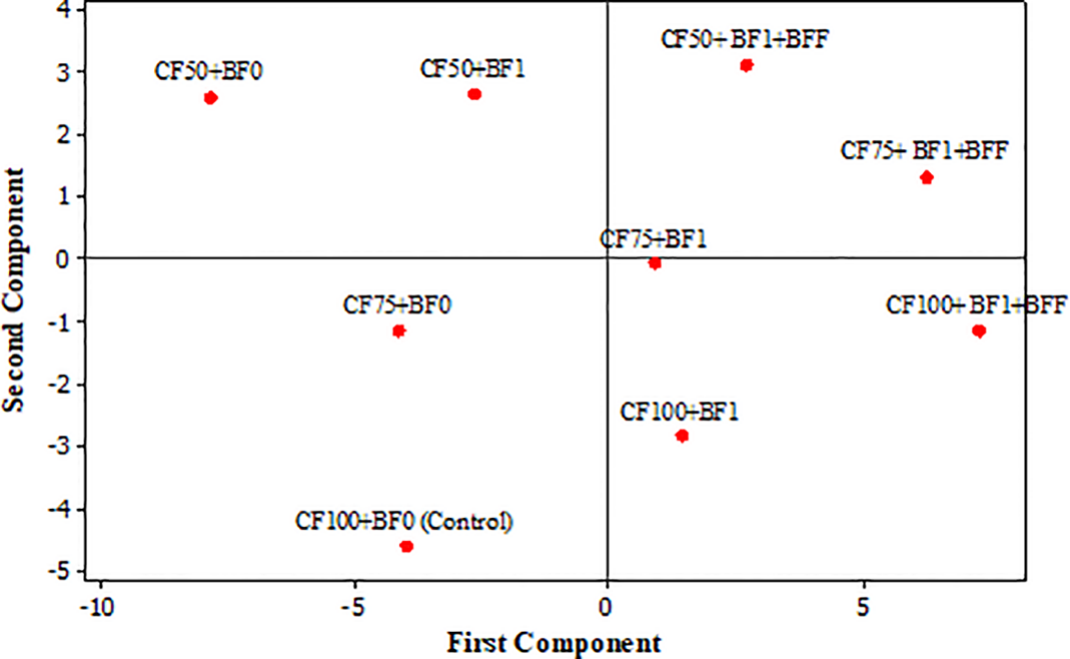
Principal component analysis (PCA) of the impacts of different treatments (Score plot) on discriminations of different determined characteristics of olive (*Olea europaea* cv. ‘Zard’). First principle components PC1 explain 70.4% and Second component PC2 explain 19.8% of the variance and distributed reproductive characteristics. Chemical Fertilizer Application (CF), was tested at three levels, 100% (CF100), 75% (CF75), and 50% (CF50) of the fertilizer requirement as determined by soil testing. This was coupled with foliar applications of a balanced 20-20-20 NPK fertilizer enriched with micronutrients. Biological Fertilizer Application (BF), also comprised three levels: BF0 (control), where trees received only soil-applied organic fertilizer without biological agents; BF1, which included a soil application of an organic fertilizer mix, mycorrhizal fungi, and the beneficial bacteria Bacillus subtilis and Pseudomonas fluorescens, supplemented with fulvic acid and amino acids; and BF1+BFF, where trees were treated with both soil and foliar applications of the aforementioned bacterial species, fulvic acid, and amino acids.

The scatter plot graph illustrates the relationship between two principal components, which are typically derived from a larger dataset spanning four years. These components serve to reduce dimensionality while still capturing the most variance. In this context, the graph provides an overview of the impacts of different treatments on the quantitative and qualitative yield of ‘Zard’ olive trees (**Figure 1**). Upon analyzing the PCA scores, a clear distinction emerges among the treatments. First Principal Component (PC1) explains approximately 70.4% of the variance. Notably, the fruit yield and characteristics such as fruit flesh thickness, fruit fresh weight, and fruit oil content are associated with specific treatments: from CF100+BF1+BFF followed by CF75+BF1+BFF and CF50+BF1+BFF in the right to CF50+BF0 treatment at the left of the score plot (**Figure 1**). Second Principal Component (PC2) is primarily related to oil quality characteristics. Specificall K_268_ and ΔK. Based on the first principal components (PC1), the optimal treatments for enhancing fruit characteristics (specifically fruit flesh thickness, fruit fresh weight, and fruit oil content) and fruit yield in ‘Zard’ olive trees were CF100+ BF1+BFF, CF75+BF1+BFF and CF50+BF1+BFF, respectively. Regarding the second principal components (PC2), which primarily relate to oil quality characteristics, specifically parameters K268 and ΔK, the best treatment options were CF50+BF1+BFF and CF50+BF1, respectively. So, the best treatments for both enhancing fruit and oil quality characteristics could be CF75+BF1+BFF and CF50+BF1+BFF, respectively.

## Discussion

Optimal growth conditions for olive trees are typically sandy, nutrient-rich substrates that allow for deep root penetration (Ozturk et al., 2021). Analytical soil assessments indicate that the soil at the research site is calcareous, exhibiting moderate salinity levels and alkaline pH. The soil profile is marked by deficient concentrations of both macro- and micronutrients, as well as organic carbon. In such calcareous soils, the bioavailability of essential nutrients like Mn, Fe, Zn, Ca, Mg, K, and P is compromised, especially when organic matter content is low, which adversely affects olive trees (Chatzistathis et al., 2010). Furthermore, an elevation in soil pH and lime content has been correlated with a considerable decline in fruit set percentage and overall yield in olive cultivations (Noori et al., 2015).

The simple effects of biological fertilizers factor revealed that the soil application of a composite organic fertilizer, mycorrhizae, and the bacteria *Bacillus subtilis* and *Pseudomonas fluorescens*, supplemented with fulvic acid and amino acids (BF1) improved leaf nutrient concentrations, but not to the extent of the CF100 control. This results in line with the results that showed the employment of plant growth-promoting rhizobacteria, *Pseudomonas putida* P159, *Pseudomonas fluorescens* T17-24, *Bacillus subtilis* P96, facilitates access to micronutrients such as zinc (Zn), iron (Fe), and phosphorus (P) in calcareous soils (Abbaszadeh-Dahaji et al., 2020). The utilization of microorganisms capable of solubilizing soil minerals within the root environment and internally within the plant can directly or indirectly enhance plant growth and development (Devi et al., 2022). The combined soil application of biological and organic fertilizers, along with foliar application of *Bacillus subtilis* and *Pseudomonas fluorescens*, fulvic acid, and amino acids (BF1+BFF), further increased nutrient concentrations, equalling or surpassing the CF100 control for N and P. this could be due to the application of amino acids that serving as the primary nitrogen source, are essential constituents during the initial phase of protein biosynthesis (Kawade et al., 2023) and can enhance the efficiency of enzymes involved in the biosynthetic pathway of fatty acids in olive trees. Amino acids also facilitate the transfer of nutrients within the trees, contributing to the sizing and coloring of the produced fruit (Cirillo et al., 2022). In addition to soil application, foliar spraying of biofertilizers has been found to be a more effective method for enhancing the nutritional status of olive trees (Sotiropoulos et al., 2024). Endophytic bacterial species of *Pseudomonas* and *Bacillus* play a pivotal role in increasing the leaf nitrogen, phosphorus, potassium, iron, and zinc. Furthermore, these bacteria are involved in the synthesis of growth stimulants such as auxin, cytokinin, and gibberellin, and in the secretion of siderophores, antibiotics, hydrogen cyanide, and volatile organic compounds (VOCs) (Sivasakthi et al., 2014).

The interaction effects of chemical and biological fertilizers demonstrated that the highest nutrient concentrations were observed when chemical fertilizer was combined with biological fertilizers (CF100+BF1+BFF and CF75+BF1+BFF), suggesting that an integrated approach to fertilization may be most beneficial for olive tree nutrition. While chemical fertilizers alone can ensure adequate nutrient supply, the integration of biological fertilizers can enhance nutrient uptake and possibly allow for a reduction in chemical fertilizer use. This is in line with recent studies, such as those by Sotiropoulos et al. (2024), which emphasize the benefits of integrating different fertilization strategies to improve olive tree yield and fruit quality. The combination of *Bacillus megaterium*, *Saccharomyces cerevisiae*, and mineral fertilizers yields a notable increase in olive trees yield parameters and nutrient content of leaves and fruits (Fawy and El-Shazly, 2016).

Our experiment demonstrated a strong positive correlation between leaf nutrient content and various fruit characteristics, including fruit length, diameter, flesh thickness, fresh weight, dry weight, and overall yield. Notably, phosphorus (P) exhibited a very strong positive correlation with fruit length (r = 0.987**) and fruit dry weight (r = 0.974**). Additionally, sulfur (S) shows a strong positive correlation with fruit fresh weight (0.986**). These findings suggest that adequate nutrient management, particularly for phosphorus and sulfur, is crucial for optimizing fruit development and yield in olive trees. In calcareous soils, where phosphorus availability is limited due to stabilization and absorption processes (Hussein et al., 2022), the use of mycorrhizal and bacterial strains can enhance phosphorus uptake (Richardson et al., 2009; Schillaci et al., 2022). Continuous phosphorus availability is critical for increasing flowering, fruit formation, and yield of olive trees (Christopoulou et al., 2021; Haberman et al., 2021; Yuan et al., 2022). Similarly, sulfur is indispensable for various biochemical reactions, including the synthesis of sulfur-containing amino acids, which are vital for protein and enzyme synthesis and plant oil production. The deficiency of sulfur or nitrogen can significantly reduce the assimilation efficiency and uptake of other nutrients, thereby affecting plant yield (Marcelić et al., 2022).

Biological fertilizers alone (BF1) improved oil quality but did not match the effectiveness of CF100. The combined foliar application of biostimulants (*Bacillus subtilis*, *Pseudomonas fluorescens*, fulvic acid, and amino acids) with BF1 (BF1+BFF) yielded fruit characteristics and oil quality comparable to CF100. This combination resulted in the highest oil content and the best quality parameters, suggesting a synergistic effect. The application of biofertilizers, both as soil application and foliar sprays, is beneficial due to their direct impact on increasing the source and sink strength in olive trees, elevating leaf nutrient concentrations, and directing photosynthetic products towards reproductive organs (Abadi et al., 2020). Research on the effects of PGPB biological stimulants (*Azetobacter crococcum*, *Bacillus subtilis*, *Bacillus megaterium*, and their combinations) applied via foliar and soil methods revealed increases in leaf chlorophyll content, photosynthesis and transpiration (Efthimiadou et al., 2020). A study investigating the combined effects of soil-applied ‘Bio Health’ biological fertilizers, foliar sprays of an amino acid solution, and boric acid on the nutritional status and performance of olive trees reported significant improvements in both the quantitative and qualitative characteristics of olive fruit (Faris and Abdulqader, 2024). Additionally, the high-quality olive oil produced by organically cultivated olive trees (Gutiérrez et al., 1999) highlights the potential of organic practices, likely including the use of biological fertilizers and biostimulants, to enhance olive oil quality.

The interaction effects of chemical and biological fertilizers, particularly when combined with foliar application of biostimulants (*Bacillus subtilis*, *Pseudomonas fluorescens*, fulvic acid, and amino acids), significantly enhances fruit characteristics and olive oil quality. This integrated approach could reduce reliance on chemical fertilizers, promoting sustainable olive farming. Application of a biofertilizer (*Nostoc muscorum*, *Anabaena oryzae*, and *Spirulina platensis*) both as a soil application and foliar spray, at varying levels of chemical fertilizer application, resulted in significant improvements growth parameters, mineral content, flowering, fruit formation, yield, and quality of olive fruit in olive trees (Mostafa et al., 2016). Also, recent studies, support the idea that combining organic and bio-fertilization strategies can enhance nutrient content of leaves, photosynthetic pigments, flowering, fruit set, fruit traits and oil quality of olive trees (Fawy and El-Shazly, 2016; Lechhab et al., 2022; Alowaiesh et al., 2023). Our results reveal that the treatments CF100+BF1+BFF, CF75+BF1+BFF, CF100+BF1, and CF50+BF1+BFF, all within the same statistical category, exhibited the highest increases in fruit water content in the fresh fruit weight, and oil content in both the fresh and dry fruit weights, compared to the control (CF100+BF0). The application of biofertilizers, especially arbuscular mycorrhizal fungi (AMF), has been recognized for improving plant water status, stomatal conductance, leaf relative water content, vegetative growth and enhancing nutrient uptake efficiency (Aganchich et al., 2022; Sharma et al., 2022). Research has also shed light on the negative impacts of abiotic stress, including water scarcity, on olive trees. Water stress has been found to affect various physiological processes such as nutrient uptake, carbon assimilation, and can lead to reduced flower and fruit setting, ultimately impacting the dry mass of the fruit and oil accumulation (Nteve et al., 2024; Sánchez-Piñero et al., 2024). Our findings emphasizing the potential benefits of combining chemical and biological fertilizers with biostimulants to achieve optimal fruit water content, olive oil quality and fruit characteristics in olive cultivation.

Full dosage of CF (CF100) yielded olive oil with optimal oxidation parameters and a balanced fatty acid profile, rich in oleic acid, which is indicative of high-quality oil with good stability. Reducing CF to 75% (CF75) increased oleic acid content, potentially enhancing the oil’s nutritional value without compromising stability. However, a 50% reduction (CF50) altered key stability indicators (K_268_ and ΔK), suggesting reduced oil stability, despite maintaining a favorable fatty acid profile. The absence of biofertilizer (BF0) resulted in the highest K_232_ values, indicative of reduced oil stability and a less desirable fatty acid profile, characterized by increased palmitic acid and decreased oleic acid levels. In contrast, the application of biofertilizer alone (BF1) enhanced both oxidation parameters and fatty acid composition, evidenced by a reduction in palmitic acid and a rise in oleic acid levels. The foliar application of biostimulants, comprising *Bacillus subtilis*, *Pseudomonas fluorescens*, fulvic acid, and amino acids, in conjunction with BF1 (BF1+BFF) yielded the most favorable oxidation parameters, as demonstrated by the lowest K_270_, K_232_, and K_262_ values, which signify heightened oil stability, and an improved fatty acid profile, with the minimal palmitic acid and maximal oleic acid content among all treatments. The interaction between CF and BF, particularly when paired with foliar biostimulants, significantly influences olive oil quality. While CF alone guarantees sufficient oil stability and an acceptable fatty acid profile, the incorporation of BF and foliar biostimulants can culminate in superior oil quality, typified by lower saturated fatty acids and higher monounsaturated fatty acids. This holistic approach may diminish the dependence on CF, fostering sustainable olive cultivation practices. Contemporary studies implying fertilization techniques, such as bio-chemical fertilizers and nanobiofertilizers, can amplify nutrient uptake, bolster oil yield and fatty acid composition, and curtail the environmental footprint of traditional fertilizers (Morales and Przybylski, 2013; Moradzadeh et al., 2021; Yadav et al., 2023). These insights corroborate our findings, underscoring the significance of a comprehensive fertilization strategy to sustain crop productivity and procure high-caliber olive oil with optimal oxidation metrics.

In our investigation, certain nutrients such as Boron (B) exhibited a distinctive correlation pattern, demonstrating a strong positive correlation with fruit oil content in fresh weight (r = 0.791**) but a strong negative correlation with free acidity (r = -0.741**). This is corroborated by research indicating that foliar application of 100 ppm boron sourced from boric acid (33.5% B) combined with 2% calcium from calcium chloride (21% Ca) twice, once at full bloom and again 15 days later, emerged as the most effective treatment for enhancing fruit set, oil content, and oil quality (Desouky, 2009). Boron treatments have been shown to increase olive fruit productivity and oil yield, leading to improved olive oil quality through enhanced fatty acid composition (Toker and Yavuz, 2015; Vishekaii et al., 2019; El-Motaium and Hashim, 2020). Moreover, foliar application of nano-boron at 20 ppm and nano-zinc at 200 ppm on Picual olive trees has been identified as the optimal treatment to achieve the highest final fruit set, resulting in the harvest of the maximum fruit yield with a high seed oil percentage and low acidity (Genaidy et al., 2020).

Our experiments indicate that higher nutrient levels generally correlate with improvements in fruit size, weight, yield, water content, and oil content. This underscores the importance of adequate nutrition, particularly Nitrogen (N), Phosphorus (P), and Potassium (K), for optimal fruit growth. Conversely, negative correlations with free acidity and peroxide value imply that nutrient management should also aim to maintain low levels of these indicators to preserve fruit quality. The differential impact on various fatty acids suggests that the effects of nutrients may vary among different fatty acid types, warranting further research into the role of balanced nutrition in fruit development and quality. Such insights could inform future agricultural practices for enhanced crop management.

Principal Component Analysis (PCA) elucidates the pivotal factors influencing the ‘Zard’ olive cultivar, offering insights to refine cultivation practices for enhanced yield and quality. The PCA indicates that leaf nutrient concentration and fruit characteristics, particularly fruit flesh thickness, fresh weight, and oil content, are the most significant factors, as reflected by their strong loadings on PC1. The subsequent components, PC2 and PC3, underscore specific oil quality attributes and certain nutrients that, while less pronounced, remain pertinent.

The variable coefficients associated with the first principal component (PC1) have revealed a significant correlation between olive fruit characteristics, namely, fruit flesh thickness, fresh weight, oil content, and yield, and the nutrient content of the leaves, emphasizing Mn, P, S, Ca, and Zn. Manganese (Mn) is an essential micronutrient for plants, playing a pivotal role in soil–plant–microbial interactions, with its availability being critical for optimal plant growth and development. Mn serves as a cofactor for several enzymes’ imperative for photosynthesis, respiration, and nitrogen metabolism activation (Thomine and Merlot, 2021). Additionally, Mn is integral to the enzyme superoxide dismutase, which safeguards plants from oxidative stress induced by reactive oxygen species. It is particularly crucial in the water-splitting complex of photosystem II (PSII) in chloroplasts, with foliar Mn levels ranging from 50 to 150 mg/kg in the youngest mature olive leaves being optimal for PSII activity (Therios, 2009; Khoshru et al., 2023).

Calcium (Ca), the most abundant element in the shoots of olive trees, is harvested annually in greater quantities than nitrogen and potassium, correlating with the yield and pruning of mature trees (Fernández-Escobar et al., 2015). Its role in alleviating environmental stress is vital, as it influences the absorption of sugars triggered by sucrose (Lecourieux et al., 2014; Handy et al., 2017). Sugar signaling acts as a mechanism for plants to assimilate various internal and external signals, thus maintaining nutrient homeostasis and modulating stress responses (Li and Sheen, 2016; Sakr et al., 2018). Desouky (2009) observed that enhancing calcium levels in olive trees improves the oil content of the fruit. Calcium is fundamental to fruit growth and development, contributing to maintaining fruit quality and firmness as a constituent of cell walls and membranes. A calcium deficiency can result in a decline in olive quality and yield. Pre-harvest foliar application of calcium can increase its concentration in the fruit, thereby improving the quality and phytochemical profile, including phenolic and bioactive compounds, in olive oil (Gouvinhas and Barros, 2020). Moreover, Hagagg et al. (2020) demonstrated that foliar application of a 0.5% calcium solution, either as calcium chelate or calcium chloride in December, enhances vegetative growth and boosts the content of iron, zinc, and manganese in olive trees.

In arid and semi-arid conditions, the accessibility of essential nutrients like calcium, boron, and zinc to young and meristematic tissues, particularly reproductive organs, is constrained (Souza et al., 2019; Wimmer et al., 2019). Therefore, foliar application of these nutrients can fulfill the increased demand by the sink (reproductive meristem organ) and ensure the availability of these elements during flower bud development.

For PC2, oil quality parameters, notably K_268_ and ΔK, emerge as influential. Leaf magnesium and nitrogen concentrations, along with other nutrients (P, K, Ca, S, Fe, Mn, Zn, Cu, B), exhibit a positive loading in PC2 (0.218) and correlate positively with oil quality traits (free acidity, peroxide value, K_270_, K_232_, K_262_, K_268_, K_274_, ΔK, fatty acid composition). These associations could potentially diminish the quality of extra virgin olive oil (Erel et al., 2013; Zipori et al., 2020). Excessive nitrogen fertilization is known to degrade olive oil quality, particularly through increased free fatty acid levels, decreased oleic acid content, and diminished oxidative stability (Fernández-Escobar et al., 2006; Dag et al., 2009; Erel et al., 2013; Pascual et al., 2019). Hence, balanced nitrogen management is essential for optimizing olive oil quality (Fernández-Escobar et al., 2006; Dag et al., 2009). For PC3, the concentration of boron (B) in leaf nutrients is identified as the most significant trait.

## Conclusions

Our study endorses a synergistic fertilization approach that integrates biological and chemical inputs to augment both the yield and quality of olive oil, alongside improving the nutritional profile of olive crops. Utilizing principal component analysis (PCA), we ascertained the most effective treatments for ‘Zard’ olive trees. The first principal component (PC1), assessing fruit characteristics such as flesh thickness, fresh weight, oil content, and overall yield, pinpointed CF100+BF1+BFF (full chemical fertilizer plus biofertilizers) and the reduced chemical fertilizer treatments CF75+BF1+BFF and CF50+BF1+BFF as the most advantageous. The second principal component (PC2), focusing on oil quality indicators like the K268 index and ΔK values, indicated that a 50% reduction in chemical fertilizer use, coupled with the application of biological fertilizers to both soil and foliage (CF50+BF1+BFF), yielded the most favorable results. A similar reduction in chemical fertilizers, with exclusive soil application of biological fertilizers (CF50+BF1), was also found to be effective. Collectively, for simultaneous improvements in fruit and oil quality, the treatments CF75+BF1+BFF and CF50+BF1+BFF are advocated, as evidenced by their placement in the first quadrant of the PCA score plot. Ultimately, the amalgamation of biological fertilizers with reduced chemical fertilizers is identified as the most superior and optimal fertilization method for the Zard cultivar in olive orchards.

## Data Availability

The original contributions presented in the study are included in the article/supplementary material. Further inquiries can be directed to the corresponding authors.

## References

[B1] Abadi V. A. Sepehri M. Rahmani H. A. Zarei M. Ronaghi A. Taghavi S. M.. (2020). Role of dominant phyllosphere bacteria with plant growth–promoting characteristics on growth and nutrition of maize (*Zea mays* L.). J. Soil Sci. Plant Nutr. 20, 2348–2363. doi: 10.1007/s42729-020-00302-1

[B2] Abbaszadeh-Dahaji P. Masalehi F. Akhgar A. (2020). Improved growth and nutrition of sorghum (Sorghum bicolor) plants in a low-fertility calcareous soil treated with plant growth–promoting rhizobacteria and Fe-EDTA. J. Soil Sci. Plant Nutr. 20, 31–42. doi: 10.1007/s42729-019-00098-9

[B3] Aganchich B. Wahbi S. Yaakoubi A. El-Aououad H. Bota J. (2022). Effect of arbuscular mycorrhizal fungi inoculation on growth and physiology performance of olive trees under regulated deficit irrigation and partial rootzone drying. S Afr J. Bot. 148, 1–0. doi: 10.1016/j.sajb.2022.03.051

[B4] Ali A. M. (2023). Establishment of nutrient sufficiency ranges in olive using boundary-line approach. J. Plant Nutr. 46, 453–461. doi: 10.1080/01904167.2022.2072335

[B5] Alonso A. Bigirumurame T. Burzykowski T. Buyse M. Molenberghs G. Muchene L.. (2016). Applied surrogate endpoint evaluation methods with SAS and R. New York: CRC Press.

[B6] Alowaiesh B. F. Gad M. M. Ali M. S. (2023). Integrated use of organic and bio-fertilizers to improve yield and fruit quality of olives grown in low fertility sandy soil in an arid environment. Phyton 0031-9457), 92. doi: 10.32604/phyton.2023.026950

[B7] Ammar G. (2024). Plant beneficial symbionts: fashionable charming members in the phytomicrobiome community. Future Perspect. Medical Pharm. Environ. Biotechnol. 1, 19–30. doi: 10.21608/FPMPEB.2024.266342.1007

[B8] Ben Salah I. Aghrouss S. Douira A. Aissam S. El Alaoui-Talibi Z. Filali-Maltouf A.. (2018). Seaweed polysaccharides as bio-elicitors of natural defenses in olive trees against *verticillium* wilt of olive. J. Plant Interact. 13, 248–255. doi: 10.1080/17429145.2018.1471528

[B9] Bhattacharyya C. Roy R. Tribedi P. Ghosh A. Ghosh A. (2020). “Biofertilizers as substitute to commercial agrochemicals,” in Agrochemicals detection, treatment and remediation (Bhattacharyya, India: Butterworth-Heinemann), pp 263–pp 290. doi: 10.1016/B978-0-08-103017-2.00011-8

[B10] Bizos G. Papatheodorou E. M. Chatzistathis T. Ntalli N. Aschonitis V. G. Monokrousos N. (2020). The role of microbial inoculants on plant protection, growth stimulation, and crop productivity of the olive tree (*Olea europea* L.). Plants 9, 743. doi: 10.3390/plants9060743 32545638 PMC7356289

[B11] Boutaj H. Meddich A. Chakhchar A. Wahbi S. El Alaoui-Talibi Z. Douira A.. (2020). Arbuscular mycorrhizal fungi improve mineral nutrition and tolerance of olive tree to *Verticillium* wilt. Arch. Phytopathol. Pflanzenschutz 53, 673–689. doi: 10.1080/03235408.2020.1792603

[B12] Brito C. Dinis L. T. Moutinho-Pereira J. Correia C. M. (2019). Drought stress effects and olive tree acclimation under a changing climate. Plants 8, 232. doi: 10.3390/plants8070232 31319621 PMC6681365

[B13] Chatzistathis T. Therios I. Alifragis D. Dimassi K. (2010). Effect of sampling time and soil type on Mn, Fe, Zn, Ca, Mg, K and P concentrations of olive (*Olea europaea* L., cv. ‘Koroneiki’) leaves. Sci. Hortic. 126, 291–296. doi: 10.1016/j.scienta.2010.07.021

[B14] Christopoulou N. Chatzistathis T. Papatheodorou E. M. Aschonitis V. Monokrousos N. (2021). The crucial role of soil organic matter in satisfying the phosphorus requirements of olive trees (*Olea europaea* L.). Agriculture 11, 111. doi: 10.3390/agriculture11020111

[B15] Cirillo A. De Luca L. Graziani G. Cepparulo M. El-Nakhel C. Giordano M.. (2022). Biostimulants application on *Olea europaea* L. @ in Mediterranean conditions increase the production and bioactive compounds of drupes and oil. Agriculture 12, 2173. doi: 10.3390/agriculture12122173

[B16] Dabbaghi O. Tekaya M. M’barki N. Ouled A. S. Öden S. Mezghani M. A.. (2018). Effect of foliar bio-fertilization on growth and biochemical parameters of olive trees at flowering. J. Plant Nutr. 41, 2281–2297. doi: 10.1080/01904167.2018.1500592

[B17] Dag A. Ben-David E. Kerem Z. Ben-Gal A. Erel R. Basheer L.. (2009). Olive oil composition as a function of nitrogen, phosphorus and potassium plant nutrition. J. Sci. Food Agric. 89, 1871–1878. doi: 10.1002/jsfa.3664

[B18] Desouky I. M. (2009). Effect of boron and calcium nutrients sprays on fruit set, oil content and oil quality of some olive oil cultivars. World J. Agri Sci. 5, 180–185.

[B19] Devi R. Kaur T. Kour D. Yadav A. Yadav A. N. Suman A.. (2022). Minerals solubilizing and mobilizing microbiomes: A sustainable approach for managing minerals’ deficiency in agricultural soil. J. Appl. Microbiol. 133, 1245–1272. doi: 10.1111/jam.15627 35588278

[B20] Efthimiadou A. Katsenios N. Chanioti S. Giannoglou M. Djordjevic N. Katsaros G. (2020). Effect of foliar and soil application of plant growth promoting bacteria on growth, physiology, yield and seed quality of maize under Mediterranean conditions. Sci. Rep. 10, 21060. doi: 10.1038/s41598-020-78034-6 33273634 PMC7713431

[B21] El-Motaium R. A. Hashim M. E. (2020). Boron efficiency in increasing olive (cv. Frantoio) fruit productivity and oil yield and quality. J. Plant Nutr. 43, 2981–2989. doi: 10.1080/01904167.2020.1806305

[B22] Ennajeh M. Ouledali S. (2024). Supplement of a commercial mycorrhizal product to improve the survival and ecophysiological performance of olive trees in an Arid region. Acta Scientiarum Polonorum Hortorum Cultus 23, 75–85. doi: 10.24326/asphc.2024.5252

[B23] Erel R. Kerem Z. Ben-Gal A. Dag A. Schwartz A. Zipori I.. (2013). Olive (*Olea europaea* L.) tree nitrogen status is a key factor for olive oil quality. J. Agric. Food Chem. 61, 11261–11272. doi: 10.1021/jf4031585 24245487

[B24] Estefan G. (2013). Methods of soil, plant, and water analysis: a manual for the West Asia and North Africa region. Third Edition. Beirut, Lebanon: International Center for Agricultural Research in the Dry Areas (ICARDA), Vol. 3. 65–119.

[B25] FAOSTAT (2023). (Food and agriculture organization of the United Nations (FAO). Available online at: http://www.fao.org/faostat/en/-data/QC (Accessed January 11, 2025).

[B26] Faris A. S. Abdulqader S. M. (2024). Response of olive trees (*Olea europaea* L.) cv. Zaity to bio health and foliar spray of tecamin max and boron. Kufa J. Agric. Sci. 16, 113–130. doi: 10.36077/kjas/2024/v16i1.10878

[B27] Fawy H. A. El-Shazly M. M. (2016). Influence of foliar applied mineral and bio-fertilizers on the yield parameters of eig and olive trees grown in the northwestern coast of Egypt. Egypt J. Soil Sci. 56, 93–112.

[B28] Fernández-Escobar R. Beltrán G. Sánchez-Zamora M. A. García-Novelo J. Aguilera M. P. Uceda M. (2006). Olive oil quality decreases with nitrogen over-fertilization. HortScience 41, 215–219. doi: 10.21273/hortsci.41.1.215

[B29] Fernández-Escobar R. Parra M. A. Navarro C. Arquero O. (2009). Foliar diagnosis as a guide to olive fertilization. Span J. Agric. Res. 7, 212–223. doi: 10.5424/sjar/2009071-413

[B30] Fernández-Escobar R. Sánchez-Zamora M. A. Garcia-Novelo J. M. Molina-Soria C. (2015). Nutrient removal from olive trees by fruit yield and pruning. HortScience 50, 474–478. doi: 10.21273/HORTSCI.50.3.474

[B31] Frangipane M. T. Costantini L. Merendino N. Massantini R. (2023). Antioxidant profile and sensory analysis in olive oils of different quality grades. Agriculture 13, 993. doi: 10.3390/agriculture13050993

[B32] Freitas J. Silva P. (2022). Sustainable agricultural systems for fruit orchards: The influence of plant growth promoting bacteria on the soil biodiversity and nutrient management. Sustainability 14, 13952. doi: 10.3390/su142113952

[B33] García-Fraile P. Menéndez E. Rivas R. (2015). Role of bacterial biofertilizers in agriculture and forestry. AIMS Bioeng 2, 183–205. doi: 10.3934/bioeng.2015.3.183

[B34] Genaidy E. A. Abd-Alhamid N. Hassan H. S. Hassan A. M. Hagagg L. F. (2020). Effect of foliar application of boron trioxide and zinc oxide nanoparticles on leaves chemical composition, yield and fruit quality of *Olea europaea* L. cv. Picual. Bull. Natl. Res. Cent 44, 1–2. doi: 10.1186/s42269-020-00335-7

[B35] Gilbraith W. E. Carter J. C. Adams K. L. Booksh K. S. Ottaway J. M. (2021). Improving prediction of peroxide value of edible oils using regularized regression models. Molecules 26, 7281. doi: 10.3390/molecules26237281 34885855 PMC8659081

[B36] Gouvinhas I. Barros A. I. (2020). “Effect of foliar pre-harvest calcium application on the mineral and phytochemical composition of olive oils,” in Proceedings, vol. 70. (Basel, Switzerland: MDPI), 66. doi: 10.3390/foods_2020-07662

[B37] Gutiérrez F. Arnaud T. Albi M. A. (1999). Influence of ecological cultivation on virgin olive oil quality. J. Am. Oil Chemists’ Soc. 76, 617–621. doi: 10.1007/s11746-999-0012-8

[B38] Haberman A. Dag A. Erel R. Zipori I. Shtern N. Ben-Gal A.. (2021). Long-term impact of phosphorous fertilization on yield and alternate bearing in intensive irrigated olive cultivation. Plants 10, 1821. doi: 10.3390/plants10091821 34579354 PMC8467881

[B39] Hagagg L. F. Merwad M. A. Shahin M. M. El-Hady E. S. (2020). Ameliorative effect of foliar application of calcium on vegetative growth and mineral contents of olive trees Kalmata and Manzanillo cultivars irrigated with saline water. Bull. Natl. Res. Cent 44, 1–6. doi: 10.1186/s42269-020-00374-0

[B40] Han Ş. Sönmez İ. Qureshi M. Güden B. Gangurde S. S. Yol E. (2024). The effects of foliar amino acid and Zn applications on agronomic traits and Zn biofortification in soybean (*Glycine max* L.). Front. Plant Sci. 15. doi: 10.3389/fpls.2024.1382397 PMC11056589 38685959

[B41] Handy G. Taheri M. White J. A. Borisyuk A. (2017). Mathematical investigation of IP 3-dependent calcium dynamics in astrocytes. J. Comput. Neurosci. 42, 257–273. doi: 10.1007/s10827-017-0640-1 28353176 PMC5756620

[B42] Herrmann L. Lesueur D. (2013). Challenges of formulation and quality of biofertilizers for successful inoculation. Appl. Microbiol. Biotechnol. 97, 8859–8873. doi: 10.1007/s00253-013-5228-8 24037408

[B43] Hussein H. A. Awad A. A. Beheiry H. R. (2022). Improving nutrients uptake and productivity of stressed olive trees with mono-ammonium phosphate and urea phosphate application. Agronomy 12, 2390. doi: 10.3390/agronomy12102390

[B44] IOC (2019). Spectrophotemtric investigation in the ultraviolet. DEC-III.4/109-VI/2019. Available online at: https://www.internationaloliveoil.org/wp-content/uploads/2019/11/Method-COI-T.20-Doc.-No-19-Rev.-5-2019-2.pdf (Accessed 3 May 2023).

[B45] Jackson J. (2016). Learn Excel Basics with Quick Examples (excel 2016, excel 2013, excel vba, Excel 2016, Excel Charts, Excel project, MS Excel, MS Excel … book, spreadsheet excel) Vol. Volume 1 (North Charleston, SC, United States: CreateSpace Independent Publishing Platform), pp 128.

[B46] Jacob S. M. Paranthaman S. (2023). Biofertilizers: An advent for eco-friendly and sustainable agriculture development. Vegetos 36, 1141–1153. doi: 10.1007/s42535-022-00550-9

[B47] Jiménez-Moreno M. J. Fernández-Escobar R. (2017). Influence of nutritional status of phosphorus on flowering in the olive (*Olea europaea* L.). Sci. Hort 223, 1–4. doi: 10.1016/j.scienta.2017.05.028

[B48] Juliano P. Gaber M. A. F. M. Romaniello R. Tamborrino A. Berardi A. Leone A. (2023). Advances in physical technologies to improve virgin olive oil extraction efficiency in high-throughput production plants. Food Eng. Rev. 15, 625–642. doi: 10.1007/s12393-023-09347-1

[B49] Kailis S. Harris D. J. (2007). Producing table olives (Australia: Landlinks press), pp 328.

[B50] Kawade K. Tabeta H. Ferjani A. Hirai M. Y. (2023). The roles of functional amino acids in plant growth and development. Plant Cell Physiol. 64, 1482–1493. doi: 10.1093/pcp/pcad071 37489637

[B51] Khoshru B. Mitra D. Nosratabad A. F. Reyhanitabar A. Mandal L. Farda B.. (2023). Enhancing manganese availability for plants through microbial potential: A sustainable approach for improving soil health and food security. Bacteria 2, 129–141. doi: 10.3390/bacteria2030010

[B52] Kiritsakis A. Keceli M. T. Kiritsakis K. (2020). “Olive oil,” in Bailey’s Industrial Oil and Fat Products. Ed. Shahidi F. (John Wiley & Sons, Toronto, ON, Canada), pp 307–pp 344.

[B53] Lechhab T. Lechhab W. Cacciola F. Salmoun F. (2022). Sets of internal and external factors influencing olive oil (*Olea europaea* L.) composition: A review. Eur. Food Res. Technol. 248, 1069–1088. doi: 10.1007/s00217-021-03947-z

[B54] Lecourieux F. Kappel C. Lecourieux D. Serrano A. Torres E. Arce-Johnson P.. (2014). An update on sugar transport and signalling in grapevine. J. Exp. Bot. 65, 821–832. doi: 10.1093/jxb/ert394 24323501

[B55] Li L. Sheen J. (2016). Dynamic and diverse sugar signaling. Curr. Opin. Plant Biol. 33, 116–125. doi: 10.1016/j.pbi.2016.06.018 27423125 PMC5050104

[B56] Maksoud M. A. Saleh M. A. El-Shamma M. S. Fouad A. A. (2009). The beneficial effect of biofertilizers and antioxidants on olive trees under calcareous soil conditions. World J. Agric. Sci. 5, 350–352.

[B57] Marcelić Š. Vidović N. Pasković I. Lukić M. Špika M. J. Palčić I.. (2022). Combined sulfur and nitrogen foliar application increases extra virgin olive oil quantity without affecting its nutritional quality. Horticulturae 8, 203. doi: 10.3390/horticulturae8030203

[B58] Marschner H. (Ed.) (2011). Marschner’s mineral nutrition of higher plants (United States: Academic press, Elsevier Science), pp 672.

[B59] Melloni R. Cardoso E. J. (2023). Microbiome associated with olive cultivation: a review. Plants 12, 897. doi: 10.3390/plants12040897 36840245 PMC9963204

[B60] Moradzadeh S. Siavash Moghaddam S. Rahimi A. Pourakbar L. El Enshasy H. A. Sayyed R. Z. (2021). Bio-chemical fertilizer improves the oil yield, fatty acid compositions, and macro-nutrient contents in *Nigella sativa* L. Horticulturae 7, 345. doi: 10.3390/horticulturae7100345

[B61] Morales M. T. Przybylski R. (2013). “Olive oil oxidation,” in Handbook of Olive Oil. Eds. Aparicio R. Harwood J. (Springer, Boston, MA). doi: 10.1007/978-1-4614-7777-8_13

[B62] Mostafa S. El-Taweel A. A. Aly A. A. (2016). Use efficiency of cyanobacteria and olive vegetation water (Cyano/Ovw) biofertilizer for olive trees under different mineral NPK levels. Egypt J. Hort 43, 77–107. doi: 10.21608/ejoh.2016.2828

[B63] Noori O. Arzani K. Moameni A. Taheri M. (2015). Vegetative growth and fruit set of olive (*Olea europaea* L. cv. ‘Zard’) in response to some soil and plant factors. J. Cent Eur. Agric. 16 (3), 319–329. doi: 10.5513/jcea.v16i3.3643

[B64] Nteve G. M. Kostas S. Polidoros A. N. Madesis P. Nianiou-Obeidat I. (2024). Adaptation mechanisms of olive tree under drought stress: The potential of modern omics approaches. Agriculture 14, 579. doi: 10.3390/agriculture14040579

[B65] Ozturk M. Altay V. Gönenç T. M. Unal B. T. Efe R. Akçiçek E.. (2021). An overview of olive cultivation in Turkey: Botanical features, eco-physiology and phytochemical aspects. Agronomy 11, 295. doi: 10.3390/agronomy11020295

[B66] Pascual M. Villar J. M. Arbones A. Rufat J. (2019). Nitrogen nutrition diagnosis for olive trees grown in super-intensive cropping systems. J. Plant Nutrit. 42(15), 1803–1817. doi: 10.1080/01904167.2019.1628983

[B67] Raimi A. Adeleke R. Roopnarain A. (2017). Soil fertility challenges and Biofertiliser as a viable alternative for increasing smallholder farmer crop productivity in sub-Saharan Africa. Cogent Food Agric. 3, 1400933. doi: 10.1080/23311932.2017.1400933

[B68] Richardson A. E. Barea J.-M. McNeill A. M. (2009). Prigent-combaret, C. Acquisition of phosphorus and nitrogen in the rhizosphere and plant growth promotion by microorganisms. Plant Soil 321, 305–339. doi: 10.1007/s11104-009-9895-2

[B69] Sakr S. Wang M. Dédaldéchamp F. Perez-Garcia M. D. Ogé L. Hamama L.. (2018). The sugar-signaling hub: overview of regulators and interaction with the hormonal and metabolic network. Inter J. Mole Sci. 19, 2506. doi: 10.3390/ijms19092506 PMC6165531 30149541

[B70] Sánchez-Piñero M. Corell M. de Sosa L. L. Moriana A. Medina-Zurita N. Madejón E.. (2024). Assessment of water stress impact on olive trees using an accurate determination of the endocarp development. Irrig Sci. 42, 461–76. doi: 10.1007/s00271-024-00914-w

[B71] Sanz-Cortés F. Martinez-Calvo J. Badenes M. L. Bleiholder H. Hack H. Llácer G.. (2002). Phenological growth stages of olive trees (*Olea europaea*). Ann. Appl. Biol. 140, 151–157. doi: 10.1111/j.1744-7348.2002.tb00167.x

[B72] Schillaci M. Raio A. Sillo F. Zampieri E. Mahmood S. Anjum M.. (2022). *Pseudomonas* and *Curtobacterium* strains from olive rhizosphere characterized and evaluated for plant growth promoting traits. Plants 11, 2245. doi: 10.3390/plants11172245 36079627 PMC9460707

[B73] Sharma A. Negi N. P. Narwal P. Kumari P. Kumar D. (2022). “Arbuscular mycorrhizal fungi: A next-generation biofertilizer for sustainable agriculture,” in Beneficial Microorganisms in Agriculture. Environmental and Microbial Biotechnology. Eds. Prasad R. Zhang S. H. (Springer, Singapore). doi: 10.1007/978-981-19-0733-3_6

[B74] Sivasakthi S. Usharani G. Saranraj P. (2014). Biocontrol potentiality of plant growth promoting bacteria (PGPR)-*Pseudomonas fluorescens* and *Bacillus subtilis*: a review. Afr J. Agric. Res. 9, 1265–1277. doi: 10.5897/ajar2013.7914

[B75] Sotiropoulos S. Chatzissavvidis C. Papadakis I. E. Kavvadias V. Paschalidis C. Antonopoulou C.. (2024). Enhancing the yield of mature olive trees via comparative fertilization strategies, including a foliar application with fulvic and humic acids, in non-irrigated orchards with calcareous and non-calcareous soils. Horticulturae 10, 167. doi: 10.3390/horticulturae10020167

[B76] Souza F. B. M. D. Coelho V. A. T. Pio R. Rodas C. L. Silva I. P. D. Melo E. T. D.. (2019). Visual symptoms and nutritional deficiencies in olive plants subjected to nutrient deprivation. Agronomy 41, e39582. doi: 10.4025/actasciagron.v41i1.39582

[B77] Tadayon M. S. Hosseini S. M. (2023). Effect of irrigation regimes and foliar nutrition on flower development and water productivity of olive (*Olea europaea* L. cv. ‘Shengeh’). J. Plant Growth Regul. 42, 735–747. doi: 10.1007/s00344-022-10580-x

[B78] Tekaya M. Mechri B. Mbarki N. Cheheb H. Hammami M. Attia F. (2017). Arbuscular mycorrhizal fungus Rhizophagus irregularis influences key physiological parameters of olive trees (*Olea europaea* L.) and mineral nutrient profile. Photosynthetica 55, 308–316. doi: 10.1007/s11099-016-0243-5

[B79] Therios I. (2009). Olives. Crop Production Science in Horticulture (Cambridge, MA, USA: CAB International), pp 425, ISBN: ISBN:9781845936204, 1845936205.

[B80] Thomine S. Merlot S. (2021). Manganese matters: feeding manganese into the secretory system for cell wall synthesis. New Phytol. 231, 2107–2109. doi: 10.1111/nph.17545 34237160

[B81] Toker C. Yavuz N. (2015). The effect of boron application on chemical characterization and volatile compounds of virgin olive oil of Ayvalik olive cultivar. J. Am. Oil Chem. 92, 1421–1428. doi: 10.1007/s11746-015-2703-7

[B82] Trolles-Cavalcante S. Y. Dutta A. Sofe,r Z. Borenstein A. (2021). The effectiveness of Soxhlet extraction as a simple method for GO rinsing as a precursor of high-quality graphene. Nanoscale Advances. 3 (18), 5292–5300. doi: 10.1039/D1NA00382H 36132643 PMC9418454

[B83] Trovato M. Funck D. Forlani G. Okumoto S. Amir R. (2021). Amino acids in plants: Regulation and functions in development and stress defense. Front. Plant Sci. 12. doi: 10.3389/fpls.2021.772810 PMC8559698 34733310

[B84] Vishekaii Z. R. Soleimani A. Fallahi E. Ghasemnezhad M. Hasani A. (2019). The impact of foliar application of boron nano-chelated fertilizer and boric acid on fruit yield, oil content, and quality attributes in olive (*Olea europaea* L.). Sci. Hort 257, 108689. doi: 10.1016/j.scienta.2019.108689

[B85] Wang D. Deng X. Wang B. Zhang N. Zhu C. Jiao Z.. (2019). Effects of foliar application of amino acid liquid fertilizers, with or without *Bacillus amyloliquefaciens* SQR9, on cowpea yield and leaf microbiota. PloS One 14, e0222048. doi: 10.1371/journal.pone.0222048 31483848 PMC6726186

[B86] Wild D. J. (2005). MINITAB release 14. doi: 10.1021/ci040130h

[B87] Wimmer M. A. Abreu I. Bell R. W. Bienert M. D. Brown P. H. Dell B.. (2019). Boron: an essential element for vascular plants. New Phytol. 226 (5), 1232–1237. doi: 10.1111/nph.16127 31674046

[B88] Yadav A. Yadav K. Abd-Elsalam K. A. (2023). Nanofertilizers: Types, delivery and advantages in agricultural sustainability. Agrochemicals 2, 296–336. doi: 10.3390/agrochemicals2020019

[B89] Yuan T. Chen S. Zhang Y. Ji L. Dari B. Sihi D.. (2022). Mechanism of increased soil phosphorus availability in a calcareous soil by ammonium polyphosphate. Biol. Fertil Soils 58, 649–665. doi: 10.1007/s00374-022-01650-z

[B90] Zipori I. Erel R. Yermiyahu U. Ben-Gal A. Dag A. (2020). Sustainable management of olive orchard nutrition: a review. Agriculture 10, 11. doi: 10.3390/agriculture10010011

